# Regulation of Thermogenic Adipocyte Differentiation and Adaptive Thermogenesis Through Histone Acetylation

**DOI:** 10.3389/fendo.2020.00095

**Published:** 2020-02-27

**Authors:** Belinda X. Ong, Reinhard Brunmeir, Qiongyi Zhang, Xu Peng, Muhammad Idris, Chungang Liu, Feng Xu

**Affiliations:** ^1^Institute of Molecular and Cell Biology, Agency for Science, Technology and Research (A*STAR), Singapore, Singapore; ^2^Department of Biochemistry, Yong Loo Lin School of Medicine, National University of Singapore, Singapore, Singapore; ^3^Cancer Science Institute of Singapore, National University of Singapore, Singapore, Singapore; ^4^Laboratory of Metabolic Medicine, Singapore Bioimaging Consortium, A*STAR, Singapore, Singapore

**Keywords:** epigenetics, gene expression, thermogenic adipocyte differentiation, adaptive thermogenesis, histone acetylation, histone deacetylation, histone acetyltransferase inhibitors, histone deacetylase inhibitors

## Abstract

Over the past decade, the increasing prevalence of obesity and its associated metabolic disorders constitutes one of the most concerning healthcare issues for countries worldwide. In an effort to curb the increased mortality and morbidity derived from the obesity epidemic, various therapeutic strategies have been developed by researchers. In the recent years, advances in the field of adipocyte biology have revealed that the thermogenic adipose tissue holds great potential in ameliorating metabolic disorders. Additionally, epigenetic research has shed light on the effects of histone acetylation on adipogenesis and thermogenesis, thereby establishing the essential roles which histone acetyltransferases (HATs) and histone deacetylases (HDACs) play in metabolism and systemic energy homeostasis. In regard to the therapeutic potential of thermogenic adipocytes for the treatment of metabolic diseases, herein, we describe the current state of knowledge of the regulation of thermogenic adipocyte differentiation and adaptive thermogenesis through histone acetylation. Furthermore, we highlight how different HATs and HDACs maintain the epigenetic transcriptional network to mediate the pathogenesis of various metabolic comorbidities. Finally, we provide insights into recent advances of the potential therapeutic applications and development of HAT and HDAC inhibitors to alleviate these pathological conditions.

## Introduction

The rising obesity epidemic constitutes one of the most pressing public healthcare issues worldwide. In the U.S., it was reported that there was an upward trend in the prevalence of obesity amongst youth and adults over the period from 1999–2000 to 2015–2016, and that the prevalence in adults reached 39.8% in 2015–2016 (1). Following this, the percentage of national medical expenditures attributed to the treatment of adult obesity grew from 6.13% in 2001 to 7.91% in 2015—a significant rise of 29% (2). Aside from the immense financial and economic burden of the modern obesity epidemic, the health implications associated with it should also not be underestimated. At present, the World Health Organization (WHO) estimates the number of overweight and obesity-related deaths to be 2.8 million per year worldwide (3). Moreover, there is accumulating evidence which indicates that obesity is an underlying risk factor for the development of metabolic disorders and chronic diseases such as type 2 diabetes (4), hypertension (5, 6), cardiovascular disease (7), as well as certain types of cancers (8, 9). Taken together, the rapid growth of global obesity and excess adiposity poses a tremendous challenge for scientists and healthcare institutions to develop successful therapeutic strategies in an effort to curb the increased mortality and morbidity derived from obesity and the comorbidities that manifest from it.

Over the past decade, there have been significant advances in the field of thermogenic adipocyte developmental biology. The thermogenic adipose tissue has been shown to hold great potential in ameliorating obesity and obesity-associated metabolic diseases (10–12). Specifically, the discovery of brown adipose tissue in adult humans (13) has greatly revised researchers' understanding of the essential role the adipose tissue plays as a critical endocrine organ involved in the regulation of metabolism and systemic energy homeostasis (14).

**Figure 1:**
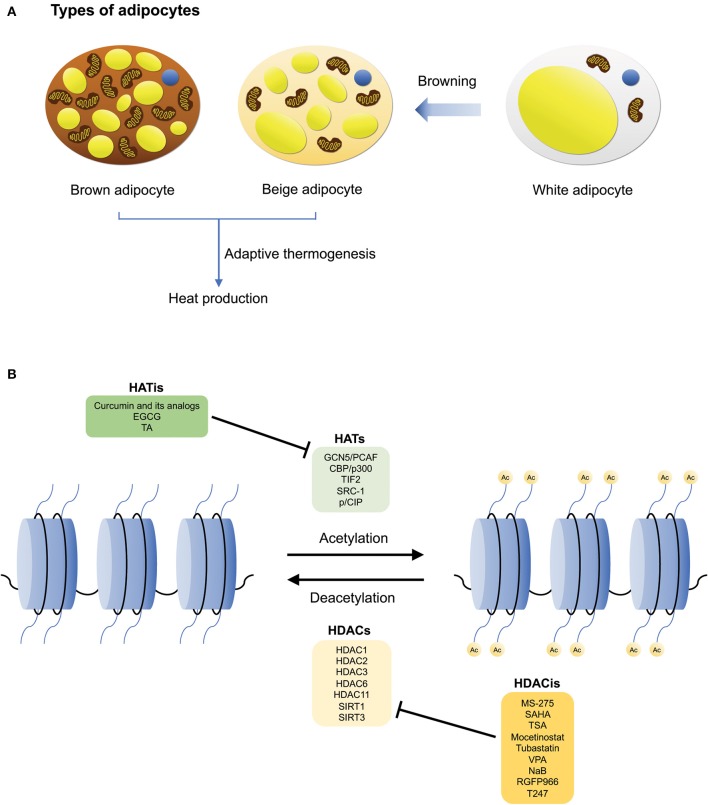
The different types of adipocytes, and HATs/HDACs that regulate brown adipogenesis and adaptive thermogenesis. **(A)** The three main types of adipocytes: white, brown, and beige adipocytes. White adipocytes are involved in energy storage, while brown adipocytes are involved in adaptive thermogenesis during which heat is produced. Brown adipocytes also consist of a high mitochondrial content and UCP1 expression. Beige adipocytes are derived from white adipocyte precursor cells and similar to brown adipocytes, they can undergo adaptive thermogenesis upon stimulation, through a process known as browning. **(B)** Histone acetylation and deacetylation are mediated by HATs and HDACs, respectively. Examples of HATs/HDACs and their inhibitors that are involved in regulating thermogenic adipocyte differentiation, adaptive thermogenesis, and the pathogenesis of metabolic disorders.

There are three main types of adipocytes found in humans—white, brown, and beige adipocytes (15) (Figure 1A). The main function of white adipose tissue (WAT) is to store energy, however, it also serves central roles in lipid storage, tissue regeneration, and the synthesis of hormones that regulate metabolic processes such as nutrient homeostasis and inflammation (15, 16). In contrast, brown adipose tissue (BAT) is involved in an energy dissipating process called adaptive thermogenesis, whereby chemical energy is converted into heat in response to environmental changes (12, 16, 17). Additionally, brown adipocytes are characterized by their high mitochondrial content and expression of uncoupling protein 1 (UCP1) (17, 18). Lastly, beige adipocytes are located within subcutaneous WAT depots, and are derived from precursor cells upon environmental cues such as cold exposure, through a process known as “browning” (16, 19, 20). Interestingly, although beige adipocytes have a lower basal expression of UCP1 relative to brown adipocytes, it has been demonstrated that their thermogenic capacity can be raised to levels comparable to that of brown adipocytes upon stimulation (19). Hence, similar to brown adipocytes, beige adipocytes also possess thermogenic properties. However, it is also notable that despite these characteristics, beige adipocytes are genetically distinct from both white and brown adipocytes (19, 21). The plasticity of the adipose tissue in mice has also been described by Vitali et al. (22). Reportedly, in C57BL/6J mice, the adipose organ consists a mixture of white adipocytes, brown adipocytes, as well as UCP1-negative multilocular adipocytes. In comparison to obesity-resistant Sv129 mice, obesity-prone C57BL/6J mice possess fewer brown adipocytes which could increase significantly upon cold acclimation (i.e., in the anterior subcutaneous and abdominopelvic depots), implicating that “browning” of white adipocytes occurred (22).

Epigenetics is defined as the study of changes in gene expression that are heritable and take place without a change in DNA sequence (23). While the association between epigenetics and metabolic diseases like hypertension (24) and diabetes (25) has been established by various studies (26–28), recent advances in epigenomic research relating to adipocyte biology have shed light on the epigenetic mechanisms affecting adipocyte differentiation and adipogenesis (29). Epigenetic modifications regulating the differentiation and function of thermogenic adipocytes remain of great interest to scientists due to their high therapeutic potential (30). In future, it is hoped that they could act as biomarkers for the diagnosis, prognosis, and prediction of metabolic diseases, and also serve as a platform for the development of drugs and therapeutic tools to alleviate these diseases.

Epigenetic modifications can be classified into four main categories: chromatin-associated modifications that include (1) DNA methylation, (2) histone modifications, and (3) chromatin remodeling (nucleosome positioning); as well as (4) non-coding RNAs (31, 32). In addition, crosstalk between different epigenetic mechanisms has also been widely investigated by researchers, substantiating the fact that certain systems do not function independently (32–35). Instead, they interact closely with one another to exert transcriptional control. A classic example which portrays how two independent epigenetic systems can be closely correlated is the association between DNA methylation and histone deacetylation. During transcriptional repression, Methyl-CpG binding (MBD) proteins (i.e., MeCP2) bind to methylated CpG and interact with histone deacetylases via their transcriptional repression domain (TRD) to trigger the deacetylation of histones (33).

Of the aforementioned epigenetics alterations, an increasing number of studies have pointed to the effects of histone modifications—in particular, histone acetylation—on adipocyte differentiation and adipogenesis (30, 36–40). Following which, in the recent years, the role of histone acetyltransferases (HATs) and histone deacetylases (HDACs) in the regulation of the development and function of thermogenic adipocytes have garnered remarkable interest from scientists and clinicians for their therapeutic potential in ameliorating obesity and its complications. In this review, we describe the current state of knowledge of the regulation of thermogenic adipocyte differentiation and adaptive thermogenesis by HATs and HDACs, along with their respective mechanisms. In addition, we highlight advances in our understanding of the therapeutic applications and development of HAT and HDAC inhibitors. Finally, we provide insights into the outlook and future perspectives of the use of these inhibitors in clinical therapies for the treatment of metabolic diseases.

## Overview of Histone Acetylation and Deacetylation

Histone acetylation and deacetylation are catalyzed by HATs and HDACs, respectively (39, 41–44). In general, multiple lysine residues on histones 3 and 4 are acetylated during transcriptional activation (i.e., H3K9, H3K14, H3K18, H3K23, H3K27, H3K56, and H4K5, H4K8, H4K12, H4K16) (40, 42, 43, 45–47). During this process, HATs catalyze the transfer of the acetyl group from acetyl-CoA onto a lysine residue (48). This leads to neutralization of the positive charge on the histone proteins and weakens their interaction with DNA (49, 50). As a result, condensed DNA (heterochromatin) loosens and becomes transcriptionally permissive in its open form (euchromatin) as it becomes accessible to DNA-binding transcription factors and RNA polymerase II (50–53). Conversely, histone acetylation can be reversed via an opposing mechanism—histone deacetylation—which involves the removal of the acetyl group by HDACs (48). When this occurs, the nucleosome becomes compacted and DNA is no longer accessible, resulting in transcriptional inhibition, and gene silencing (53, 54).

On the whole, HATs can be classified into three major families: the GNAT superfamily, MYST family and p300/CBP family (55). Apart from this classification, HATs can also be further characterized based on their cellular localization (i.e., in the nucleus or cytoplasm) (45). On the other hand, HDACs are categorized into four different classes: the zinc-dependent classes include class I (HDAC1-3 and 8), class IIa (HDAC4, 5, 7, and 9), class IIb (HDAC6 and 10) and class IV (HDAC11); and the NAD^+^-dependent class consists of class III (SIRT1-7) (56, 57).

Because histone acetylation modulates a myriad of cellular and metabolic pathways, a dysregulation of the expression and activities of HATs and HDACs have been implicated in various pathological conditions including cancers (52, 58–60), neurological disorders (50, 61–63), autoimmune diseases (54, 64–67), and in particular relevance to this review, obesity, and its metabolic comorbidities such as inflammation, insulin resistance, and liver steatosis (25). Given their importance in regulating gene expression and their potential as attractive therapeutic targets for the treatment of metabolic diseases, several key HATs, and HDACs have been identified by researchers for their involvement in adipogenesis (39). This review will focus only on those that are involved in the regulation of thermogenic adipocyte differentiation, adaptive thermogenesis and the pathogenesis of metabolic disorders (Figure 1B).

## Histone Acetyltransferases

In general, the HAT families are classified according to the sequence similarity of their catalytic HAT domain (68). However, each family also possesses distinct structural features and catalytic mechanisms that distinguish them from one another.

### Structural Features and Catalytic Mechanisms

**Figure 2:**
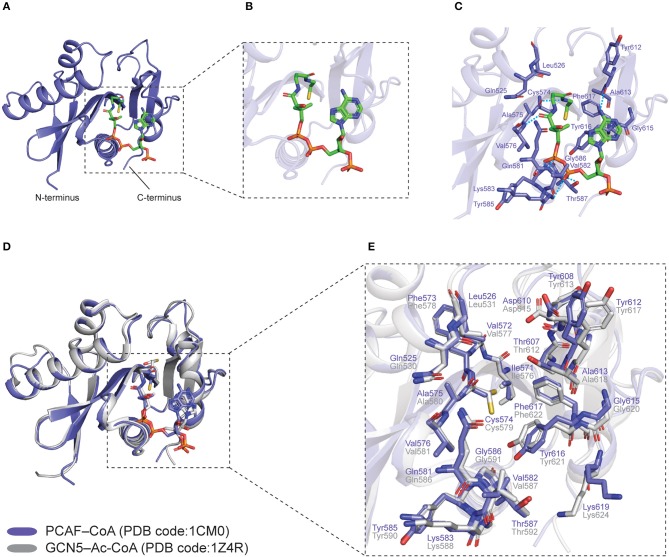
Co-crystal structures of human PCAF–CoA (PDB code: **1CM0**) and GCN5–Ac-CoA (PDB code: **1Z4R**) HAT domains. PCAF–CoA is shown in purple, while GCN5–Ac-CoA is shown in gray. **(A)** Structure of PCAF–CoA comprising a central core composed of a β-sheet flanked by a parallel α-helix on one side, as well as α and β segments surrounding it. CoA is shown as green sticks. **(B)** Magnified view of the CoA molecule in a bent conformation. **(C)** Interaction between PCAF residues and CoA at the catalytic site. Hydrogen bonds are shown as blue dashed lines. **(D)** Superimposition of PCAF–CoA and GCN5–Ac-CoA overall structures. CoA and Ac-CoA are shown as sticks in the same color as their corresponding HAT proteins. **(E)** Superimposition of PCAF–CoA and GCN5–Ac-CoA residues at the catalytic site. CoA and Ac-CoA have been removed for clarity. All structural figures were produced using *PyMOL* (The PyMOL Molecular Graphics System, Version 2.3.2 Schrödinger, LLC) (69).

The overall structure of HATs consists of a structurally conserved central core which comprises a β-sheet that is made up of three β-strands, flanked by a parallel α-helix on one side (68, 69) (Figure 2A). The core region is further surrounded by structurally distinct α and β segments that differ across the HAT families, all in all, forming a substrate-binding pocket that facilitates enzyme-substrate interactions (68, 70) (Figure 2A).

On the whole, proteins in the GNAT superfamily are characterized by a catalytic HAT domain consisting of ~160 residues, and a bromodomain located at the C-terminus that targets acetylated lysine (71). Interestingly, in spite of the low sequence homology, a conserved core fold is observed amongst members of the family (72). The common fold is made up of six-seven β-strands and four α-helices (β0-β1-α1-α2-β2-β3-β4-α3-β5-α4-β6), spanning four conserved motifs in the following order: C-D-A-B, with motifs A and B, in particular, mediating binding of the acceptor substrate and acyl-CoA (73). Contrastingly, MYST proteins not only contain a HAT domain that is made up of ~250 residues, many of them also possess a chromodomain and a zinc-binding domain located at the N-terminus of the enzyme and within the HAT domain, respectively (71). Finally, in comparison to GNAT and MYST proteins, the ~500-residue HAT domain within the p300/CBP family proteins is distinctively larger; moreover, similar to the MYST family, the structure of p300/CBP proteins also comprises of other conserved domains such as the bromodomain and the zinc-binding TAZ, PHD, and ZZ domains that facilitate interaction with other proteins (71).

More importantly, each family has a unique mechanism to catalyze the transfer of the acetyl group. The GNAT superfamily (i.e., Hat1/KAT1, GCN5/KAT2A, PCAF/KAT2B) utilizes a ternary complex mechanism, through which both its N- and C-termini facilitate histone substrate binding; the MYST family (i.e., MOF/KAT8/MYST1, TIP60/hKAT5, HBO11/MYST2/KAT7) utilizes a ping-pong mechanism that involves autoacetylation of a specific lysine at the catalytic site for cognate histone acetylation; and last but not least, the p300/CBP family (i.e., P300/KAT3B, CBP/KAT3A) utilizes a hit-and-run mechanism, where an autoacetylation loop and a substrate-binding loop are also essential for maximal enzymatic activity as well as binding of acetyl coenzyme A and lysine, respectively (70).

### Overview of Metabolic Homeostasis Through Histone Acetylation

Several *in vivo* studies have substantiated the association between aberrant histone acetylation and metabolic complications. Mikula et al. showed that levels of histones H3K9 and H3K18 acetylation at two key inflammatory mediator genes, *Tnfa* and *Ccl2*, are upregulated in obese mice (74). In another study, it was revealed that the level of H3K9 (and to a lesser extent H3K14) acetylation at TNF-α and COX-2 promoters in monocytes is significantly augmented in diabetic patients (75). Similarly, in the context of epigenetic machinery-related gene expression, *lysine acetyltransferases* (human HATs) and *Bromodomain-containing protein 2* expressions were found to be elevated (in contrast to the decreased expression of most *HDACs*) in mice fetal liver and placental labyrinth tissues of fetuses affected by maternal obesity (76). Likewise, Dorneles et al. demonstrated that hyperacetylation of H4, accompanied by a decrease in HDAC2 activity, was observed in lipopolysaccharide (LPS)-stimulated peripheral blood mononuclear cells (PBMC) of obese males after strenuous exercise (77). Collectively, these studies highlight the importance of histone acetylation on the regulation of metabolic homeostasis.

## Regulation of Thermogenic Adipocyte Differentiation by HATs

In the following section, we will examine how some HATs regulate the transcriptional control of BAT development.

### GCN5/PCAF

**Figure 3:**
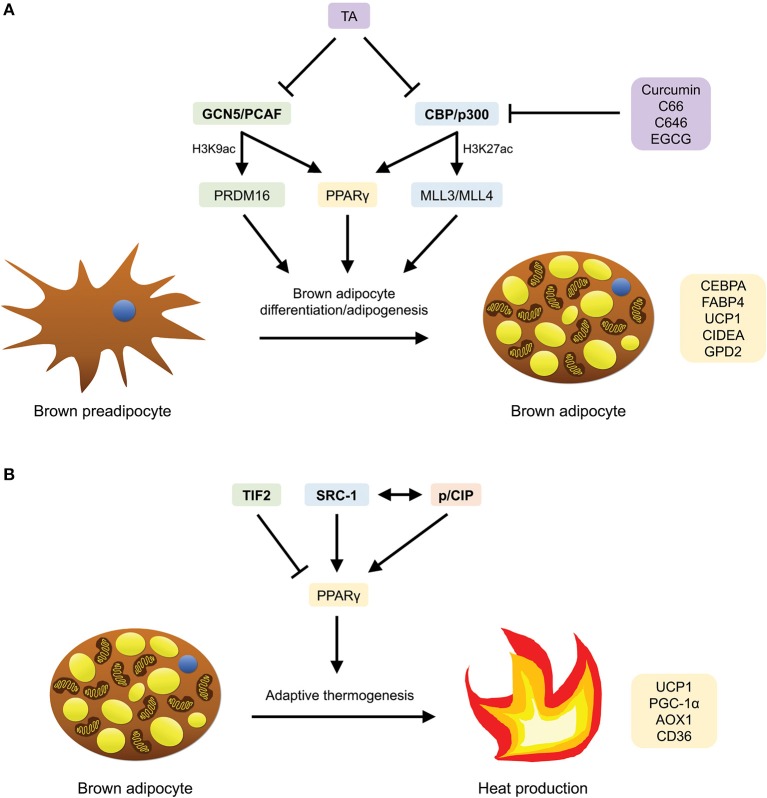
HATs that are involved in brown adipocyte differentiation/adipogenesis and adaptive thermogenesis, as well as compounds (HATis) that have been demonstrated to inhibit them. **(A)** GCN5/PCAF and CBP/p300 mediate brown adipocyte differentiation/adipogenesis by inducing the expression of PPARγ-target, BAT-selective, thermogenic and adipogenic genes through PRDM16 and MLL3/MLL4, respectively, as well as PPARγ. **(B)** TIF2, SRC-1 and p/CIP mediate adaptive thermogenesis by inducing the expression of BAT-specific PPARγ-target genes. SRC-1 and p/CIP have also been shown to interact with each other to regulate the expression of these genes.

The HATs GCN5 (Kat2a) and its homolog PCAF (Kat2b) induce gene activation through the acetylation of histone H3K9 (78, 79). The level of H3K9ac has been reported to increase during adipogenesis (80). In a study conducted by Jin et al. to investigate the function and mechanisms by which GCN5 and PCAF mediate adipogenesis, it was revealed that GCN5 and PCAF act redundantly to modulate the expression of adipogenic genes such as PPARγ, as well as the BAT transcription factor PR-domain–containing 16 (PRDM16) (79) (Figure 3A). Reportedly, *Gcn5/PCAF* double knockout (DKO) cells showed a reduction of H3K9ac in brown preadipocytes and inhibition of adipogenic gene expression, while *Gcn5*^flox/flox^;*PCAF*^−/−^;*Myf5-Cre* mice displayed defects in BAT development (79). Furthermore, the authors also demonstrated through DKO cells that GCN5/PCAF not only function upstream of PPARγ to control PPARγ expression, but are also essential for the expression of *Prdm16* (via the recruitment of Pol II onto the *Prdm16* gene) during brown adipogenesis (79). Since PRDM16 is a predominant regulator for BAT development, taken together, these findings suggest a regulatory role of GCN5/PCAF in the transcriptional control of BAT development and brown adipocyte differentiation.

Crystal structures of the HAT domain of human GCN5 and PCAF, bound to acetyl coenzyme A (Ac-CoA) and coenzyme A (CoA), respectively, have been solved by three groups [GCN5–Ac-CoA, PDB code: **1Z4R** (81); PCAF–CoA, PDB code: **1CM0** (82), **4NSQ** (83)]. Specifically, in the PCAF–CoA complex structure, it can be observed that the CoA molecule is in a bent conformation (Figure 2B), and interacts with the protein predominantly through its pantetheine arm and pyrophosphate group (82) (Figure 2C). Superimposition of the overall structures of GCN5–Ac-CoA (**1Z4R**) with PCAF–CoA (**1CM0**) reveals the high structural similarity between the two paralogs, with a root-mean-square deviation (r.m.s.d.) of 0.373 Å (Figure 2D). Moreover, catalytic residues at the substrate binding pocket around the central core domain (83) are also highly conserved and adopt almost identical orientations (Figure 2E). These findings provide an explanation for the overlapping roles of the two homologs from a structural perspective.

### CBP/p300

The CREB-binding protein (CBP) and E1A-binding protein (p300) acetylate histones H3K18 and H3K27 in mammalian cells (39). Recently, through epigenomic profiling, Lai et al. have demonstrated that during brown adipogenesis, CBP/p300 bind and activate enhancers through a process mediated by H3K4me1/2 methyltransferases MLL3/MLL4 (84) (Figure 3A). Specifically, it was observed that along with MLL4, CBP identifies super-enhancers (SEs) involved in adipogenesis (SEs of the genes *Pparg, Cebpa*, and *Fabp4*), and that MLL3/MLL4 are essential for the formation of SEs (84). In addition, an increase in enhancer-binding by CBP was found to be accompanied by an increase in the level of H3K27ac and vice versa. More significantly, the authors showed that MLL4 recognizes primed SEs of several BAT-selective genes (*Ucp1, Cidea*, and *Gpd2*) that are involved in thermogenesis in BAT (84). To summarize, this study has elucidated the central roles which MLL3/MLL4 (enhancer-priming) and CBP/p300 (enhancer-activation) play in adipogenesis and brown adipocyte differentiation.

Intriguingly, it has also been unraveled through ribozyme-mediated targeting of CBP/p300 in 3T3-L1 preadipocytes that a downregulation of the expression of either CBP or p300 alone corresponds with a decrease in the expression of PPARγ-target genes and suppression of adipocyte differentiation (85). This implies that in spite of the high sequence similarity between them, these paralogous proteins do not fully complement each other in the regulation of adipocyte differentiation (85). Therefore, in future, it would be interesting to unravel the potential functional differences between CBP and p300, in regard to the authors' hypothesis that the two enzymes may act at different points during the process of adipogenesis (85).

**Figure 4:**
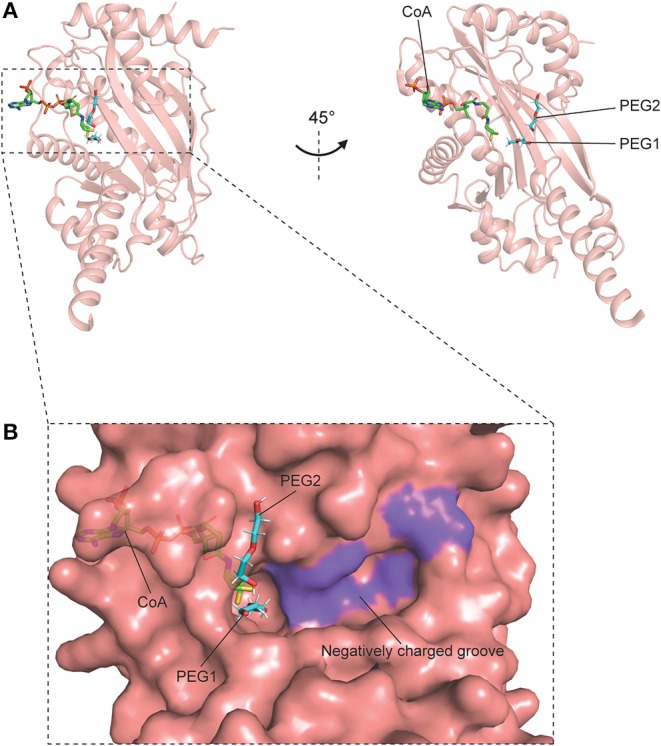
Crystal structure of p300 catalytic domain in complex with CoA, containing PEG1 and PEG2 (PDB code: **4PZR**). p300 is shown in pink, CoA is shown as green sticks, and the PEG moieties are shown as cyan sticks. **(A)** Overall structure of p300–CoA catalytic domain comprising a central β-sheet and α-helices around it. **(B)** Surface of the catalytic site and the region surrounding it. The shallow, negatively charged groove composed of Ser1396, Tyr1397, Thr1357, and Asp1625 is colored in purple.

Structural studies on human p300 have been extensively carried out by several groups in the recent years. In earlier studies, crystal structures of p300 in complex with the synthetic bi-substrate inhibitor lysyl CoA (Lys-CoA) were determined: HAT domain–Lys-CoA (PDB code: **3BIY**) (86); bromo-RING-PHD-HAT domains–Lys-CoA [PDB code: **4BHW** (87), **6GYR** (88)]. Structural analyses and mutational studies of these co-crystals further highlighted the crucial roles of two residues, Trp1436 and Tyr1467, in the regulation of the catalytic activity of the enzyme (86–89). Following this, the complex structures of p300 bound to Ac-CoA, acyl-CoA, and their variants were also elucidated (68, 89). In particular, the structures of the p300 HAT domain comprising its substrate Ac-CoA (PDB code: **4PZS**), product CoA (PDB code: **4PZR**) and inhibitor acetonyl-CoA (PDB code: **4PZT**) were reported by Maksimoska and coworkers in a study (89). Upon superimposition with the HAT domain–Lys-CoA structure, the authors noted a similar overall fold and conformation amongst the structures, including a characteristic central β-sheet made up of nine α-helices encompassing seven β-strands (Figure 4A). Interestingly, it was also observed that the HAT domain–CoA structure contains two PEG moieties (PEG1 and PEG2) located near the co-factor binding site (89). This discovery, along with the identification of a shallow, negatively charged, potential substrate-binding groove (Ser1396, Tyr1397, Thr1357, and Asp1625) by the same group in a previous study, implicates a peptide path for the association between the enzyme and its substrates—through which the substrate could track between PEG2 and the negatively charged groove (86, 89). Taken together, these findings provide structural rationales for the design of novel small molecule p300 inhibitors that could target these newly identified substrate-binding sites with increased selectivity and specificity (Figure 4B).

## Regulation of Adaptive Thermogenesis by HATs

In the following examples, we will examine how co-regulators, which are known to interact with nuclear receptors (on top of their intrinsic HAT activity), affect adaptive thermogenesis and energy homeostasis. In particular, we will look at three members from the p160 family: the transcription intermediary factor 2 (TIF2), steroid receptor coactivator 1 (SRC-1) and p/CIP (90–92).

### TIF2

In a study conducted by Picard et al. to investigate the homeostatic effects of TIF2 in two obesity models (high-fat feeding and neonatal injection of monosodium glutamate), the authors discovered that in the absence of TIF2, mice are resistant to the development of obesity (91) (Figure 3B). Specifically, the ablation of TIF2 resulted in enhancements in metabolic profiles and adaptive thermogenesis. The improvements in metabolic parameters include higher insulin sensitivity (lower fasting glycemia and triglyceride levels; higher glucose clearance and uptake rates), increased lipolysis, as well as diminished fat uptake and storage capacity, most likely due to a loss of PPARγ activity (91). The upregulation of thermogenic activity observed in BAT of TIF2^−/−^ mice was mirrored by changes to the morphology of BAT, together with an increase in oxygen consumption, mRNA expression of UCP1, PGC-1α, and acetylCoA oxidase (AOX), rectal temperature as well as the levels of plasma free fatty acid and circulating ketone bodies upon cold exposure and fasting (91). Collectively, these findings illustrate that in TIF2^−/−^ mice, there was an augmentation of thermogenic activity, coupled with an increase in the capacity for energy expenditure through higher fatty acid oxidation and respiration uncoupling.

### SRC-1

In the same study conducted by Picard and coworkers, it was further unraveled that in contrast to the absence of TIF2, the lack of SRC-1 renders mice susceptible to obesity (91) (Figure 3B). Reportedly, SRC-1 and TIF2 compete for PGC-1α and the formation of the PPARγ/PGC-1α complex (91). Moreover, particularly, the lack of TIF2 results in a more active SRC-1/PGC-1α complex. This contributes in part to the enhanced thermogenic profiles in the mice, thereby implicating that an alteration in the ratio of TIF2/SRC-1 in diet-induced obesity modifies the energy balance in BAT, with implications in the pathogenesis of obesity. Strikingly, in contrast to the absence of TIF2, SRC-1^−/−^ mice displayed diminished energy expenditure and impaired adaptive thermogenesis, as well as other metabolic changes including increased lipid infiltration in BAT, lower rectal temperature upon cold exposure and fasting, reduced fatty acid oxidation, lower oxygen consumption, decreased mRNA expression of UCP1, PGC-1α, and AOX—all in all, resulting in higher body weight gain and an obese phenotype (91).

### p/CIP

The essential role SRC-1 plays in regulating energy balance, along with another nuclear receptor co-activator, p/CIP, is further highlighted in a study performed by Wang et al., where *p/CIP*^−/−^*/SRC-1*^−/−^ mice were shown to display defective adaptive thermogenic activity (92) (Figure 3B). In the paper, the researchers revealed that the absence of both *p/CIP* and *SRC-1* suppressed the development of BAT and the expression of several PPARγ target genes in BAT which are essential for BAT development, mitochondrial uncoupling and adaptive thermogenesis (*CD36, AOX1, UCP1*) (92). Furthermore, when fed a high-fat diet (HFD) and exposed to cold, the double knockouts exhibited lower body temperature, accompanied by decreased lipid storage and expression of *UCP1* in brown fat, thereby indicating an impairment in their adaptive thermogenic response. However, despite these findings, an increase in the basal metabolic rates and levels of physical activity were observed in these mice—therefore, ultimately, rendering them lean and resistant to HFD-induced obesity (92).

## HAT Inhibitors

Given their clinical significance and therapeutic potential in attenuating obesity and related metabolic disorders, the identification and development of compounds which regulate the activity of HATs have been explored by scientists over the years (although notably, research on the development of HAT inhibitors is not as extensive as compared to that of HDAC inhibitors). In the following section, we will examine some HAT inhibitors that have been shown to mediate the amelioration of metabolic comorbidities (Figure 1B).

### Curcumin and Its Analogs

The HAT inhibitor (HATi) curcumin (diferuloylmethane), which is found in the spice turmeric (*Curcuma longa*), was first demonstrated in 2004 to specifically inhibit the HAT activity of p300/CBP (but not of PCAF) (93) (Figure 3B). The therapeutic potential of curcumin has been investigated in relation to obesity, diabetes, insulin resistance, and obesity-associated inflammation. For example, in HFD-induced obese and diabetic mice fed with curcumin, an amelioration of diabetes (in the context of glycemic status and insulin sensitivity) and obesity-associated inflammation was observed, together with decreased NF-kB activity in the liver, reduced macrophage infiltration into WAT and elevated adiponectin production (94). Similarly, in another study, mice administered with long-term oral supplementation of curcumin were shown to be resistant to the development of HFD-induced obesity and insulin resistance via the suppression of the inflammatory response in adipocytes and hepatic lipogenesis (95).

More recently, novel curcumin analogs and derivatives have been identified for their potential use as anti-diabetic agents (i.e., for kidney disease). In particular, the curcumin analog C66 has been shown to mitigate diabetic nephropathy in diabetic mice through the suppression of c-Jun N-terminal kinase (JNK) activation, p300/CBP expression and total HAT activity (96). In another study examining the epigenetic effects of glucose on the expression of the proinflammatory gene, thioredoxin-interacting protein (TXNIP), in diabetic Sur1-E1506K^+/+^ mice, it was discovered that the p300 inhibitor C646 reverses histone acetylation and consequently, represses hyperglycemia-stimulated *TXNIP* expression (97) (Figure 3B).

### Epigallocatechin-3-Gallate

Found as the major polyphenol in green tea, the HATi epigallocatechin-3-gallate (EGCG), which has been found to inhibit p300/CBP-mediated acetylation of p65 *in vitro* and *in vivo* (98), has been implicated as an anti-obesity agent in several studies (99–101) (Figure 3B). In one of the earliest studies, the role of EGCG in attenuating obesity was established by Klaus and colleagues through dietary supplementation with EGCG in HFD-induced obese mice models (99). Experimental results showed that chronic administration of EGCG over a 4-week period led to a dose-dependent reduction in body weight gain and fat accretion in obese male mice. In addition, the following changes in gene expression were observed: in WAT—decreased leptin and stearoyl-CoA desaturase-1 (SCD1) gene expression; in the liver—decreased SCD1, malic enzyme (ME), and glucokinase (GK) gene expression, as well as a dose-dependent increase in UCP2 gene expression, possibly resulting from an enhancement of fatty acid oxidation (99) [SCD1, ME, and GK are involved in the synthesis of monounsaturated fatty acids, lipid synthesis and liver glycolysis, respectively (99)].

In a subsequent study using HFD-induced obese C57BL/6J mice to investigate how EGCG regulates the thermogenic activity of BAT and hypothalamic inflammation, it was found that the development of diet-induced obesity was prevented upon 4 week dietary EGCG supplementation (100). In particular, experimental findings revealed an increase in the mRNA expression of BAT mitochondrial biogenesis and thermogenesis genes (*Ucp1, Pgc-1*α, and *Prdm16*), a rise in body temperature upon cold exposure, as well as a decrease in body weight gain, blood glucose and total triglyceride levels, adipose tissue lipid accumulation and microglia-mediated inflammation in the hypothalamus (via the inhibition of NF-κB and STAT3 pathways) (100). Together, these results show that EGCG led to an enhancement of BAT thermogenesis and amelioration of neuroinflammation in obese mice.

In a more recent discovery, Li et al. have provided new insights into the mechanisms by which EGCG decreases the susceptibility of male C57BL/6J mice to HFD-induced obesity (101). Reportedly, the intragastric administration of 100 mg/kg EGCG over the course of 20 weeks resulted in a significant increase in the activation of AMPK in the subcutaneous and epididymal adipose tissue of the mice. This finding is accompanied by a decrease in epididymal adipose tissue weight gain, triglycerides, cholesterol, and low-density lipoprotein cholesterol (LDL-C) levels, as well as an increase in high-density lipoprotein cholesterol and fecal free fatty acids levels, and the mRNA expression of lipolysis and fatty acid oxidation genes in WAT (101). In summary, the authors showed that EGCG alleviates hyperlipidemia in mice through AMPK activation to a certain extent.

### Tannic Acid

Tannic acid (TA) is a representative of gallotannins—a major component of hydrolysable tannins—that is found in a wide range of foods and beverages of plant origin, such as fruits and vegetables (102, 103). The polyphenolic compound has been implicated as an anti-obesity, anti-diabetic (for type 2 diabetes), anti-lipogenic and anti-adipogenic agent in several studies (103–105). For example, in 3T3-L1 cells, TA was shown to induce insulin-mediated glucose transport (via phosphorylation of the insulin receptor and Akt, and translocation of GLUT4) and suppress adipocyte differentiation (via a downregulation in the expression of the adipogenic gene *PPAR*γ and changes in the expression of cell proliferation genes *c-fos, c-jun*, and *c-myc*) (103). Subsequently, it was established by Nie et al. that TA exerts its anti-adipogenic effect by inhibiting the expression of PPARγ, specifically at the early stage of differentiation of 3T3-L1 preadipocytes (105).

Intriguingly, it was not until recently that the role of TA as a novel HATi was confirmed by Chung et al. through *in vitro* and *in vivo* non-alcoholic fatty liver disease (NAFLD) models, where TA was found to effectively inhibit the activities of p300, CBP, and PCAF; in particular, it inhibits p300 with the highest efficiency at an IC_50_ value of 3.886 mM (106) (Figures 3A,B). Further *in vitro* experiments using oleic and palmitic acid (OPA)-treated HepG2 cells revealed that TA reduced OPA-induced lipid accumulation via the downregulation of the mRNA expression of genes associated with lipogenesis (*ACLY, FASN, SREBP-1c*, and *PPAR*γ). Additionally, through *in vivo* experiments where C57/BL6 mice were fed a Western diet (WD) consisting of 1 or 3% TA for 12 weeks, it was revealed that mice fed with 3% TA supplementation had reduced body weight and lipid accumulation (in the liver) that is comparable to that of the control group, as well as a significant decrease in triglyceride, total cholesterol, and LDL-C levels. Likewise, TA was also shown to suppress HAT activity and the mRNA expression of lipogenic genes *in vivo* (106). Notably, the anti-HAT activity of TA was also associated with hypoacetylation of histones H3K9 and H3K36. Collectively, through *in vitro* and *in vivo* models, the authors demonstrated that TA supplementation alleviated the pathogenesis of NAFLD, thereby establishing both the anti-HAT and anti-lipogenic properties of TA.

In the same study, through p300–TA docking simulations, the authors revealed a model illustrating the interaction between p300 and TA (106). In the structure, TA lies in a catalytic cleft located between the bromo- and RING domains of p300, and interacts with p300 through 13 hydrogen bonds. As a consequence, the authors proposed that TA diminishes the catalytic activity of p300 by stimulating a conformation change of the enzyme (106). These findings potentially provide the structural basis to develop novel TA derivatives which inhibit the HAT activity of p300 or its paralogs by targeting potential catalytic residues at the bromo- and RING domains.

## Histone Deacetylases

HDACs are involved in a variety of biological processes and they regulate various cellular functions such as cell proliferation, differentiation, grow arrest, cell death (including apoptosis, autophagy, and mitotic cell death) as well as gene expression (107). Because of their essential role in maintaining cellular homeostasis, aberrant expression and alterations to the activity of HDACs are frequently associated with the pathogenesis of several diseases such as cancers, neurodegenerative diseases, and pulmonary diseases (107, 108). Furthermore, loss-of-function studies have also reported that deletion of HDACs can lead to developmental defects such as cardiac malformation, gastrulation defects, and endothelial dysfunction in knockout mice (56).

### Structural Features and Catalytic Mechanisms

HDACs are classified into four classes according to their sequence homology. Class I (HDAC1-3 and 8), class IIa (HDAC4, 5, 7, and 9), class IIb (HDAC6 and 10) and class IV (HDAC11) HDACs contain zinc-binding domains, and thus, fall under the canonical zinc-dependent group of HDACs (56, 57). In general, class I HDACs are located mainly in the nucleus, while class II HDACs (other than HDAC10) are able to shuttle between the nucleus and cytoplasm, although they are mainly found in the cytoplasm (40, 107). The former is usually associated with cellular proliferation and survival, while the latter seems to have more tissue-specific roles (107). Lastly, class IV HDACs and HDAC10 are localized in the nucleus, even though it has also been reported that HDAC11 co-precipitates with HDAC6, which is found in the cytoplasm (40, 109). In contrast, class III HDACs (SIRT1-7) constitute the NAD^+^-dependent family of proteins known as sirtuins, which are structurally and functionally distinct from zinc-dependent HDACs (56, 57).

**Figure 5:**
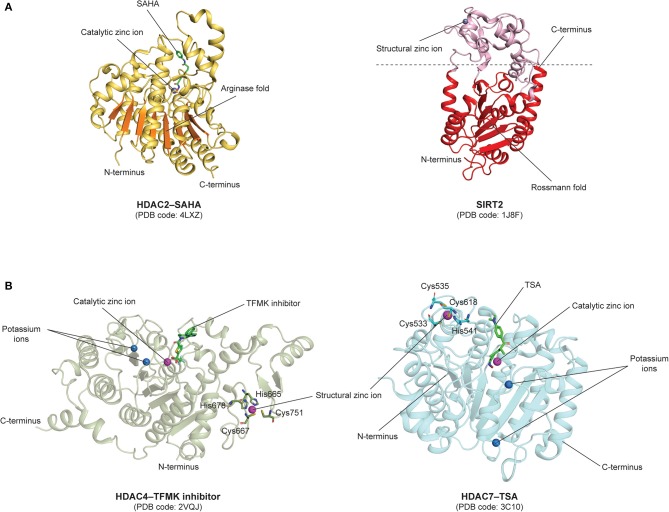
Structural features of human HDACs. **(A)** Zinc- and NAD^+^-dependent classes HDACs: Catalytic domains of zinc-dependent HDAC2–SAHA (PDB code: **4LXZ**) comprising an arginase fold colored in orange, and NAD^+^-dependent SIRT2 (PDB code: **1J8F**) comprising a Rossmann fold colored in red. **(B)** Class IIa HDACs: HDAC4–TFMK inhibitor (PDB code: **2VQJ**) and HDAC7–TSA (PDB code: **3C10**) structures comprising a catalytic zinc-binding domain and an additional structural zinc-binding domain. The structural zinc ions are coordinated by histidine and cysteine residues shown as sticks.

Notably, a prominent structural distinction among the different classes of HDACs is that members from the zinc-dependent classes are characterized by a classical arginase fold, while those from the NAD^+^-dependent class exhibit a typical Rossmann fold, a structural motif that is usually found in nucleotide-binding proteins (110, 111). The arginase and Rossmann folds are depicted in the HDAC2 (PDB code: **4LXZ**) (112) and SIRT2 (PDB code: **1J8F**) (113) structures, respectively (Figure 5A). Notably, the arginase fold contains a central β-sheet consisting of eight parallel β-strands (111), while the Rossmann fold in SIRT2 contains a β-sheet made up of six parallel β-strands and six alternating α-helices (113).

Other structural features of class I HDACs include a catalytic site that comprises two His residues, one Tyr residue, a nucleophilic water, a zinc binding domain with an Asp/Asp/His triad and a potassium ion located away from the catalytic zinc ion (110).

**Figure 6:**
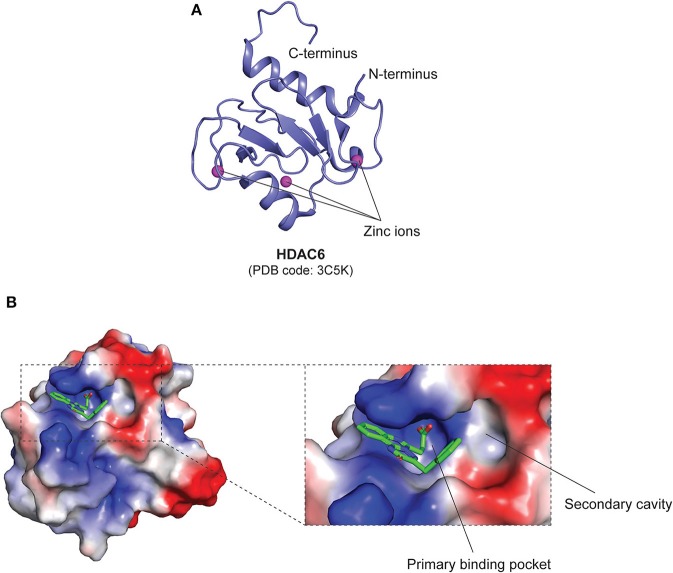
Structural features of the class IIb HDAC, HDAC6 ZnF-UBD. **(A)** Overall structure of the ZnF-UBD of HDAC6 (PDB code: **3C5K**). **(B)** Surface representation of inhibitor-bound human HDAC6 ZnF-UBD (PDB code: **5WBN**). The electrostatic potential map shows the inhibitor-bound primary binding pocket, and the opening of a secondary cavity that can be potentially targeted to increase inhibitor selectivity. Basic, acidic and hydrophobic regions are shown in blue, red, and white, respectively. The inhibitor is shown as green sticks.

In class IIa HDACs, the catalytic Tyr residue is substituted to a His residue, rendering them as weak deacetylases (110). Apart from the catalytic zinc-binding domain, class IIa HDACs also contain a structural zinc-binding domain that is only conserved in class IIa HDACs and not found in other classes, as seen in the structures of trifluoromethylketone (TFMK) inhibitor-bound HDAC4 (PDB code: **2VQJ**) (114) and TSA-bound HDAC7 (PDB code: **3C10**) (115) (Figure 5B). The structural zinc ion in HDAC4 is coordinated by His665, Cys667, His678, and Cys751 (114), while the structural zinc ion in HDAC7 is coordinated by Cys533, Cys535, His541 and Cys618 (115) (Figure 5B). Also, unlike class I HDACs which possess short N- and C-terminal extensions, class IIa HDACs possess long N-terminal extensions that mediate the binding of the transcription factor myocyte enhancer factor 2 (MEF2) and the chaperone protein 14-3-3 (56). On the other hand, Class IIb HDACs are structurally similar to class I HDACs, with minor differences in regard to their catalytic mechanisms. It has also been revealed that the catalytic His residues can act as general base and acid (110). Furthermore, it is also worthy to note that, intriguingly, HDAC6 is composed of two HDAC domains and a zinc-finger ubiquitin-binding domain (ZnF-UBD) at the C-terminus, making it structurally different from the rest of the HDACs (PDB code: **3C5K**) (56, 116, 117) (Figure 6A).

Similar to class I and II HDACs, the class IV HDAC, HDAC11, also harbors a homologous HDAC domain, and short N- and C-terminal extensions (56).

Lastly, the NAD^+^-dependent class III HDACs only function in the presence of NAD^+^, through which a ternary complex is formed between the deacetylase and the substrate (110). As such, sirtuins have been implicated in the fluctuation of intracellular NAD^+^ concentration during metabolic regulation, for instance, through the activation of the salvage pathway or upon nutritional stimuli (118). Notably, the dual role of class III HDACs allows them to act as both a deacetylase and a ADP ribosyl transferase where they mediate the transfer of the acetyl group to ADP-ribose after nicotinamide is released from NAD^+^. Additionally, similar to class IIb HDACs, a conserved His residue that functions as the general base is also found in class III HDACs (110).

## Regulation of Adaptive Thermogenesis and Browning by HDACs

### HDAC1 and HDAC2

**Figure 7:**
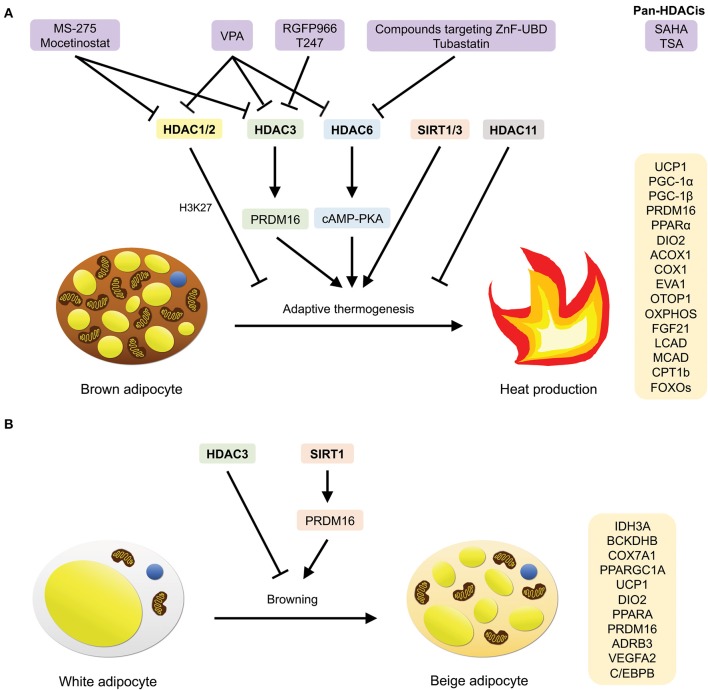
HDACs that are involved in adaptive thermogenesis and browning, as well as compounds (HDACis) that have been demonstrated to inhibit them. **(A)** HDAC1/2/3/6/11 and SIRT1/3 mediate adaptive thermogenesis by inducing the expression of BAT-specific, thermogenic, lipid β-oxidation, and transcription regulation-related genes. **(B)** HDAC3 and SIRT1 mediate browning of white adipocytes by inducing the expression of oxidative metabolic genes and classical BAT markers.

A recent study conducted by Li et al. has shed light on the role which HDAC1 plays in the negatively regulation of the thermogenic program in mature brown adipocytes (119) (Figure 7A). Suppression of *Hdac1* promotes acetylation and prevents methylation of histone H3K27, resulting in an increase in the expression of BAT-specific genes. In the paper, the authors first reported that the protein level of HDAC1 was prominently higher in WAT as compared to BAT; and upon cold exposure or injection with a β_3_-adrenergic agonist, the expression level of *Hdac1* in BAT of A/J mice was found to decrease. It was further shown in BAT1 cells that knockdown of *Hdac1* significantly elevates BAT-specific gene expression (*Ucp1, Pgc-1*α, *Pgc-1*β, *Prdm16, Ppar*α, *Dio2, Acox1, Cox1, Eva1, Otop1*) and isoproterenol-stimulated UCP1 protein expression levels (119). In contrast, overexpression of *Hdac1* was shown to diminish BAT-specific gene expression. Notably, the authors also observed similar findings in HIB-1B cells. HDAC1 dissociation from the promoters of the BAT-specific thermogenic genes, *Ucp1* and *Pgc-1*α, was subsequently observed in BAT of mice and differentiated BAT1 cells upon sympathetic activation with a β_3_-adrenergic agonist or isoproterenol. Along with these results, enhancement of H3K27 acetylation and reduction in H3K27 trimethylation were observed in isoproterenol-treated and/or *Hdac1*-knockdown BAT1 cells (119). Taken together, these findings established the role of HDAC1 in downregulating the transcriptional activation of BAT-specific thermogenic genes and consequently, impairing the thermogenic program in brown adipocytes.

In another study, through quantitative phosphoproteomics, along with genetic and pharmacological inhibitory methods, Shinoda and colleagues have identified the serine/threonine kinase, Casein Kinase 2 (CK2), as a negative regulator of the cAMP-mediated thermogenic program in white adipocytes and biogenesis of beige adipocytes in mice (120). Upon the inhibition of CK2 *in vivo*, the authors observed a rise in energy expenditure, as well as alleviation of HFD-induced obesity and insulin resistance via the induction of UCP1-mediated thermogenesis. More importantly, it was revealed that inhibition of CK2 diminishes CK2-dependent phosphorylation of HDAC1 (Ser393, 421, and 423) and HDAC2 (Ser422 and 424), and increases *Ucp1* expression, stimulating the thermogenic program in white adipocytes (120). In sum, these data provide insights into how class I HDACs contribute to the action of the CK2 inhibitor on thermogenesis in white adipocytes through the enhancement of thermogenic gene expression.

### HDAC3

Besides HDAC1/2, another class I HDAC, namely HDAC3, has also been implicated in the thermogenic activity of BAT, WAT and beige fat located in inguinal white adipose tissue (iWAT) (121–123).

BAT-specific genetic ablation of HDAC3 resulted in the susceptibility of B6 mice to cold exposure due to an impairment in their ability to maintain core body temperature, leading to severe hypothermia and subsequently, death (121) (Figure 7A). Moreover, the absence of HDAC3 in BAT was also shown to impede metabolic respiration (decreased noradrenaline-induced oxygen consumption) and mitochondrial respiration (presence of large lipid droplets). In association with these findings, the loss of BAT-specific HDAC3 also caused a near-complete downregulation of the expression of both the *Ucp1* gene and UCP1 protein at thermoneutrality, as well as a decrease in the expression of genes involved in mitochondrial oxidative phosphorylation (OXPHOS) (121). Lastly, through global run-on sequencing (GRO-seq) and genome-wide *de novo* motif analysis, a crucial role for HDAC3 as coactivator of estrogen-related receptor α (ERRα) was discovered. Reportedly, HDAC3 induces the basal transcription of BAT-specific thermogenic genes (*Ucp1, Ppargc1a*, and OXPHOS genes) by deacetylating PGC-1α (121). Collectively, these results demonstrate how HDAC3 mediates the maintenance of thermogenic gene networks to regulate the thermogenic capacity of BAT, which may be stimulated upon acute to extreme cold exposure.

Alternatively, HDAC3 has been shown to partake in a strikingly different role in WAT. In contrast to its role in BAT, Ferrari and co-authors reported that HDAC3 controls the metabolic phenotype of WAT by functioning as a molecular brake to impede WAT browning (122) (Figure 7B). Selective deletion of *Hdac3* in adipose tissues led to alterations in the phenotype and metabolic profile of iWAT, and notably, not BAT. These changes include the reddish appearance of iWAT, as well as an increase in hematoxylin-eosin staining, UCP1 staining and the number of multilocular adipocytes. More significantly, a rise in body temperature upon cold exposure, as well as an upregulation of oxidative metabolic (*Idh3a, Bckdhb, Cox7a1*, and *Ppargc1a*) and classical BAT marker (*Ucp1, Dio2, Ppara, Prdm16, Adrb3*, and *Vegfa2*) gene expression was also observed. This indicates that the absence of *Hdac3* promoted browning and elevated the thermogenic activity of WAT (122). On top of this, it was revealed that the loss of *Hdac3* in adipocytes stimulates a futile cycle of fatty acid metabolism which produces Ac-CoA, thereby promoting an increase in the acetylation of histone H3K27 on *Pparg* and *Ucp1* enhancers, as well as putative regulatory regions of *Ppara in vitro* and *in vivo*—the mechanism that is ultimately responsible for determining the browning and thermogenic capacity of WAT (122).

In another study conducted by Liao et al., the researchers discovered that HDAC3 is involved in PRDM16-mediated thermogenesis in brown and beige adipocytes (123) (Figure 7A). PRDM16 is known as an essential BAT transcription factor that regulates brown and beige adipose function. The researchers first revealed in the study that the protein levels of HDAC3 are lower in BAT as compared to iWAT, and in response to cold exposure, a decrease in HDAC3 protein levels was observed in both adipose tissues, suggesting that thermogenesis may be induced through the inhibition of HDAC3. Further experiments using human and murine adipocyte cultures treated with HDAC3 inhibitors confirmed that upon HDAC3 inhibition, there was an increase in the expression of thermogenic genes (*Ppargc1a, Ucp1*, and *Fgf21*), and henceforth, the thermogenic activity of both brown and beige adipocytes (123). Interestingly, it was also unraveled that PRDM16 interacts with HDAC3. Genetic ablation of *Prdm16* in primary brown adipocytes isolated from BAT, as well as primary brown and inguinal adipocytes isolated from *Prdm16-null* mice displayed a decrease in inhibitor-stimulated thermogenic gene expression, especially *Ucp1*, indicating that PRDM16 contributes to HDAC3-mediated thermogenesis (123).

### HDAC6

Apart from Class I HDACs, class IIb HDACs have also been linked to the regulation of thermogenesis in BAT. It was reported by Jung et al. that HDAC6 knockout mice exhibited lower interscapular BAT depot surface temperature and rectal temperature, as well as a reduction in the mRNA and protein expression levels of UCP1—altogether, signifying a deficiency in the thermogenic activity of BAT (124) (Figure 7A). Upon further investigation, it was found that in correlation with defective thermogenesis, brown adipocytes isolated from BAT of knockout mice also displayed lower levels of cAMP and UCP1 protein expression (which could be restored with 10 μM forskolin treatment). This implies that cAMP-PKA signaling was suppressed in knockout mice and that the inhibition led to the repression of UCP1 expression. Additionally, lipid accumulation (larger brown adipocytes and lipid droplets) was also observed in BAT of the knockout mice, further suggesting that a deficit in thermogenic response resulted in impaired lipid utilization in HDAC6-deficient mice. In summary, the study showed that HDAC6 regulates BAT thermogenesis by activating the cAMP-PKA signaling pathway to stimulate UCP1 expression (124).

### HDAC11

The lone member of class IV HDACs, HDAC11, was recently identified as a regulator of metabolic homeostasis (125). HDAC11-deficient mice were shown to be resistant to HFD-induced obesity and the metabolic syndrome (Figure 7A). In the study, the researchers observed a decrease in body weight gain, epididymal white adipose tissue (eWAT) weight and the size of adipocytes found in eWAT of HFD-fed knockout mice. Additionally, hypercholesterolemia, glucose intolerance, insulin resistance, and hepatic steatosis were also ameliorated in these mice. More significantly, the knockout mice exhibited higher body temperature upon cold exposure, as well as higher UCP1 mRNA and protein expression levels in BAT in response to HFD treatment. Therefore, these data indicate that there was an overall enhancement of thermogenesis in the HDAC11 knockout mice (125).

Another study found that loss of HDAC11 in mice leads to an increase in the formation of BAT [higher interscapular BAT (iBAT) mass] as well as beiging of WAT (higher abundance of multilocular adipocytes, thermogenic gene expressions of *Ucp1* and *Pgc-1*α, and UCP1 protein expression upon cold exposure) (126) (Figure 7A). Similar to the previous study, knockout mice also displayed enhanced cold-induced thermogenic potential (higher body temperature, as well as *Ucp1* and *Pgc-1*α expression, and smaller lipid droplets in iBAT). In addition, upon HFD treatment, it was observed that there was an increase in fatty acid oxidation (elevated oxygen consumption, metabolic rate, and total energy expenditure), improved glucose tolerance and insulin sensitivity, as well as an attenuation of obesity and hepatic steatosis. Furthermore, knockdown of HDAC11 also enhanced brown adipocyte differentiation in mouse embryonic fibroblasts (MEFs), resulting in an upregulation of *Ucp1* and *Pgc-1*α mRNA and protein expression levels. The authors also unraveled that HDAC11 represses brown adipocyte differentiation and thermogenic gene expression by interacting with BRD2, thereby suggesting that this domain could potentially be targeted in future for the selective inhibition of HDAC11 (126).

### SIRT1

The NAD^+^-dependent class III HDACs, which comprises of members from the sirtuin family, have also been implicated in the regulation of adaptive thermogenesis. Particularly, in the recent years, SIRT1 was reported to induce the browning of WAT (127) and also enhance BAT function, including its thermogenic potential (128).

Qiang and co-authors uncovered that Prdm16 specifically interacts with deacetylated Pparγ, which is mediated by SIRT1 (on Lys268 and Lys293), and that SIRT1 gain-of-function resulted in the upregulation of BAT-selective gene expression (*Ucp1*) and the downregulation of WAT-selective gene expression involved in insulin resistance (*Agt, Chemerin, Pank3, Resistin*, and *Wdnm1L*) *in vitro* (127) (Figure 7B). Consistent with these findings, in response to cold-induced thermogenesis, mice with enhanced SIRT1 activity (Dbc1^−/−^ mice) exhibited an increase in the abundance of Ucp1-immunohistochemical stained paucilocular iWAT and the expression of brown-selective genes (*Ucp1* and *C/ebp*β), along with a decrease in the expression of white-selective genes (*Chemerin* and *Resistin*), implying that browning occurred in white adipocytes. Likewise, upon chronic exposure, glucose tolerance was restored in aging Dbc1^−/−^ mice with insulin resistance. In mice overexpressing SIRT1 (*SirBACO* mice), lower weight gain and higher expression of brown, angiogenic, and lipolytic genes were observed in iWAT in response to chronic exposure, while a decrease in WAT-selective insulin resistance-associated genes was observed upon high-fat feeding (127). Collectively, these findings illustrate the simultaneous brown remodeling and enhancement of WAT function when SIRT1 is overexpressed.

More recently, it was demonstrated that in homozygous transgenic mice (SIRT1^Tg/Tg^), moderate overexpression of SIRT1 improved glucose homeostasis due to an enhancement of BAT thermogenic activity (128) (Figure 7A). Interestingly, the mice displayed higher energy expenditure, glucose tolerance and insulin sensitivity through an increase in BAT glucose uptake, even when fed a low fat diet (LFD). Upon cold exposure, higher body temperature, which correlates to an enhancement of BAT thermogenic activity, was observed. Further analyses revealed that SIRT1^Tg/Tg^ BAT exhibited higher mRNA expression of key genes involved in lipid β-oxidation (*LCAD, MCAD*, and *CPT1b*) and transcription regulation (*PPARa* and *FOXOs*), as well as an increase in BAT-selective gene expression (*Ucp1* and *Dio2*), UCP1 protein expression, uncoupled respiration, citrate synthase activity and lipolysis (128). These results indicate an enhancement of BAT thermogenic function in SIRT1^Tg/Tg^ mice, and henceforth, establish SIRT1 as a critical regulator of thermogenesis and glucose homeostasis in BAT.

### SIRT3

Besides SIRT1, SIRT3 has also been revealed by Shi et al. to be highly expressed in BAT as compared to WAT; and in response to cold exposure, the RNA expression of SIRT3, along with UCP1, was elevated, suggesting a potential role for SIRT3 in modulating cold-induced thermogenesis (129) (Figure 7A). Constitutive expression of SIRT3 was found to increase the RNA expression of both UCP1 and PGC-1α, as well as mitochondria-related genes. Since UCP1 can be activated by PGC-1α upon cold exposure, through SIRT3 mutational studies, the authors discovered that UCP1 expression can be restored upon coexpression of PGC-1α with a SIRT3 mutant. This indicates that PGC-1α regulates the function of SIRT3 in promoting UCP1 expression, and that SIRT3 promotes mitochondrial electron transport activity and uncoupling capacity. Consistent with these findings, the authors further showed that in obese mice, a reduction of SIRT3 RNA expression was coupled with downregulated RNA expression of UCP1 and mitochondria-related genes (129). In summary, the study identified SIRT3's role in regulating adaptive thermogenesis through the augmentation of mitochondrial respiration.

## HDAC Inhibitors

As mentioned earlier, in comparison to HATis, research on HDAC inhibitors (HDACis) is much more extensive, and includes a wide range of HDACis – from global HDACis (pan-HDACis) to those that target only certain classes of HDACs (selective HDACis). In the following section, we will look at how some HDACis regulate gene expression, adipocyte differentiation, adaptive thermogenesis and the development of metabolic comorbidities.

### MS-275, SAHA, and TSA

MS-275 is a class I HDACi known for its inhibitory properties against HDAC1 and HDAC3, while SAHA is a pan-HDACi (119, 130–132) (Figure 7A). When C2C12 myotubes were treated with these inhibitors, mitochondrial biogenesis, and oxidative metabolism were stimulated (130). Additionally, *in vivo*, class I HDAC inhibition resulted in a remarkable decrease in body weight, while both global and class I HDAC inhibition led to a decrease in fasting glycemia, circulating insulin, and triglycerides, as well as improvements in glucose tolerance, insulin resistance and hepatic steatosis in obese diabetic *db/db* mice. Of note, increases in energy expenditure and heat production were also observed in the mice upon MS-275 treatment; and in skeletal muscle, a notable increase in oxidative metabolism was observed (higher expression of mitochondrial function-, glucose and lipid metabolism-, TCA cycle- and oxidative phosphorylation-related genes) upon global and class I HDAC inhibition. Moreover, in the BAT of these mice, HDAC inhibition by SAHA and MS-275 led to a decrease in brown adipocyte cell size and an increase in iBAT. More importantly, MS-275 inhibition resulted in higher body temperature upon cold exposure, upregulated expression levels of BAT markers (*Ucp1, Elovl3, Dio2, Cidea, Prdm16*, and *Adrb3*) and genes involved in mitochondrial biogenesis. Likewise, *in vitro*, in response to SAHA or MS-275 treatment, differentiated primary brown adipocytes also displayed higher expression of BAT markers and mitochondrial biogenesis-related genes. On the other hand, in WAT, class I inhibition induced the browning of WAT by promoting the expression of BAT-associated genes, especially *Ucp1*. Lastly, the study showed that class I HDAC inhibition stimulates PGC-1α expression by preventing the binding of HDAC3 onto the promoter. This in turn, promotes the expression of oxidative markers in BAT and skeletal muscle, and brown remodeling of WAT (130). In sum, the authors revealed that MS-275 and to some extent, SAHA, alleviates obesity and diabetes by enhancing the thermogenic and oxidative metabolic capacity of skeletal muscle and BAT, promoting the browning of WAT.

Subsequently, in a murine model of diet-induced obesity, the same group of researchers demonstrated that MS-275 stimulated the browning of visceral and subcutaneous WAT in HFD-fed mice (131). Notably, thermogenic capacity was enhanced in response to acute cold exposure, and the expression of *Ucp1* and other BAT markers were upregulated in WAT. Additionally, the mice exhibited body weight loss, improved glucose tolerance, as well as higher expression of genes involved in adipose tissue functionality, lipolysis and fatty acid β-oxidation (131). In a more recent study, the authors further delineated that MS-275 induces browning through an epigenetic imprinting mechanism that remodels the metabolic phenotype of white adipocytes during the early stages of differentiation (132). When C3H/10T1/2 cells were treated with MS-275 during differentiation, there was an increase in the expression of browning markers and genes related to oxidative metabolism. This was accompanied by an increase in H3K27 acetylation at enhancer regions of *Pparg* and *Ucp1*, resulting in higher expression of these genes (132). The findings presented by these studies thus help to further shed light on the role of MS-275 in governing the adipose cell fate and potentiating the oxidative and browning capacity of white adipocytes (131, 132).

In another study conducted by Li et al., which was examined earlier on in this review, the authors demonstrated the effects of MS-275, SAHA and another pan-HDACi, trichostatin A (TSA) (119) (Figure 7A). Reportedly, MS-275 elevated the expression of BAT-specific genes (*Ucp1, Elovl3*, and *Pgc-1*α) induced by isoproterenol in BAT1 cells. Furthermore, combined treatment of both MS-275 and *Hdac1* siRNA knockdown did not result in any additive effects on *Ucp1* and *Pgc-1*α expressions. Thus, it was proposed that *Hdac1* mediates BAT function by acting as a molecular target of MS-275. In contrast, interestingly, SAHA and TSA inhibited isoproterenol-induced expression of BAT-specific genes (*Ucp1, Prdm16, Ppar*γ and *Otop1*), while promoting the expression of mitochondria oxidation-related genes (*Pgc-1*α and *Acox1*). These results suggest that unlike MS-275, which enhances BAT-specific gene expression in BAT1 cells, SAHA regulates BAT function through a more complex mechanism, potentially involving the activity of other HDAC family members (119).

Lastly, because SAHA represses the activity of class I and class II HDACs—and with the latter being mainly involved in cell differentiation—it was hypothesized that the different roles which class I and class II HDACs play may have resulted in the difference in gene expressions when pan- and class I inhibitors were used separately (119). Nevertheless, since the effects of MS-275 were more pronounced, it can be deduced that class I HDACis hold therapeutic potential in enhancing BAT function and alleviating metabolic disorders.

### Mocetinostat and Tubastatin

As mentioned earlier, Shinoda et al. have uncovered that HDAC1 is involved in CK2-related white adipocyte thermogenesis (120). CK2 inhibition was shown to induce UCP1-mediated thermogenesis by attenuating CK2-dependent phosphorylation of HDAC1 and upregulating *Ucp1* expression. Addition of the class I HDACi, Mocetinostat, to inguinal white adipocytes treated with the highly selective CK2 inhibitor CK2-VIII, blocked its effect on *Ucp1* and *Elovl3* expression (Figure 7A). When the cells were treated with the class II HDAC6-selective inhibitor, Tubastatin (which does not interact with CK2 in adipocytes), CK2-VIII-induced *Ucp1* and *Elovl3* expression levels remain unaffected (120). These data suggest that class I HDAC inhibition, coupled with CK2 inhibition, prevents the enhancement of thermogenesis in white adipocytes.

### Valproic Acid

Valproic acid (VPA) is a class I and II HDACi that has recently been revealed to ameliorate insulin resistance, fat accumulation and gluconeogenesis in type-2 diabetes-induced rats (133) (Figure 7A). Khan et al. discovered that in diabetic rats, body weight gain, BAT fat deposition, hepatic steatosis, islets beta-cell damage, plasma glucose and HbA1c levels, and insulin resistance were blunted in response to VPA treatment (133). Furthermore, hepatic histone H3ac levels were elevated, and AKT-2 and GSK3β phosphorylation levels in the liver were restored, thereby suggesting that VPA decreases plasma glucose levels by promoting glucose conversion into glycogen. The mRNA expression of *Foxo1, G-6-pc*, and *Pdk-4*, as well as FOXO1 nuclear translocation and glucagon expression were also reduced. Of note, the study also revealed that treatment with the anti-diabetic drug metformin yielded comparable results with these data. Collectively, these findings indicate that VPA mediates glycogen synthesis, regulates the insulin/AKT signaling pathway, and inhibits glucagon- and FOXO1-linked gluconeogenesis, and therefore, holds potential to be repurposed as an anti-diabetic therapeutic agent (133).

### Sodium Butyrate

The short-chain fatty acid HDACi, sodium butyrate (NaB), was also demonstrated by the same group of researchers in another study to improve glucose homeostasis in type-2 diabetes-induced rats through the attenuation of insulin resistance, fat accumulation, dyslipidemia and gluconeogenesis (134). It was observed that hepatic steatosis and fat accumulation, as well as islets damage significantly decreased upon NaB treatment. This was accompanied by hypertrophy of adipocytes in WAT and BAT, and an increase in adipocyte abundance/unit area in the diabetic rats. NaB also suppressed the increase in total hepatic HDAC activity and restored the decrease in histone H3ac levels due to diabetes. Moreover, the researchers noted a reduction in plasma glucose and HbA1c levels, a decrease in insulin resistance, and an elevation of AKT-2 phosphorylation level. Along with these findings, nuclear translocation of FOXO1 and glucagon expression were also found to be reduced, signifying that gluconeogenesis was inhibited in NaB-treated diabetic rat (134). Most notably, these findings are comparable to the results collected from rats treated with metformin (134). Overall, the study shows that through HDAC inhibition, NaB improved diabetes-associated pathologies in diabetic rats, and therefore sheds light on the anti-diabetic and beneficial effects of NaB.

The positive regulation of butyrate on insulin sensitivity in HFD-fed mice was also revealed in an earlier study conducted by Gao et al. (135). NaB supplementation led to higher energy expenditure and fatty acid oxidation, a reduction in fasting blood glucose and insulin levels, improved intraperitoneal insulin tolerance, enhanced BAT thermogenic activity upon cold exposure, upregulation of UCP-1 and PGC-1α mRNA expression, as well as an increase in type I skeletal muscle fibers, Additionally, there was a loss of 10.2% in body weight and 10% in fat content in the diet-induced obese mice model—all in all, signifying that administration of NaB led the mice to become resistant to obesity and insulin resistance (135).

### RGFP966 and T247

As mentioned previously, Liao and colleagues have identified HDAC3 as a negative regulator of PRDM16-mediated thermogenesis in brown and beige adipocytes (123). In the same study, the researchers found that in response to the highly selective HDAC3 inhibitor, RGFP966 (RGFP), thermogenic gene expression (*Ppargc1a, Ucp1*, and *Fgf21*) was upregulated in primary brown adipocytes, primary brown and inguinal adipocytes isolated from mice, immortalized brown adipocytes and C3H-10T1/2 cells, and differentiated human adipose stem cells (hASCs) (Figure 7A). In agreement with these findings, a rise in the levels of acetylated lysine in C3H-10T1/2 cells were also observed. Importantly, treatment with another highly selective HDAC3 inhibitor, T247, yielded comparable results—with a similar increase in both thermogenic gene expression and acetylated lysine levels in immortalized brown adipocytes. Furthermore, using *Prdm16* knockdown brown adipocytes, as well as primary brown and inguinal adipocytes isolated from *Prdm16-null* mice, it was shown that the increase in thermogenic gene expression due to RGFP, especially *Ucp1*, was blunted. This suggest that PRDM16 may function downstream of HDAC3 to mediate thermogenesis, and that through a fat cell-autonomous mechanism, pharmacological inhibition of HDAC3 enhances the thermogenic potential of brown and beige adipocytes irrespective of their genetic makeup (123).

## Conclusions and Future Perspectives

In the current review, we have described how the regulation of histone acetylation and deacetylation affects thermogenic adipocyte differentiation and the thermogenic potential of adipose tissues. In particular, the studies mentioned provide crucial insights into how HATs and HDACs maintain the epigenetic transcriptional network by regulating the expression of a plethora of genes, including thermogenic genes, adipocyte differentiation markers, and adipose tissue-specific genes. These findings thus shed light on the mechanisms that mediate the pathogenesis of various metabolic comorbidities.

In addition, we have also covered recent advances in regard to the use of novel small molecules, the repurposing of several drugs and the development of therapeutic agents as potential strategies to control the expression and activities of HATs and HDACs. Henceforth, it is hoped that in future, these pharmaceutical compounds—HATis and HDACis—would serve as anti-obesity and anti-diabetic drugs in clinical therapies to counteract the development of pathological conditions such as obesity, glucose intolerance, insulin resistance, and hepatic steatosis for the treatment of metabolic diseases. Moreover, they could also be used to promote favorable cellular and metabolic conditions such as WAT browning, BAT development and enhance the thermogenic capacity of beige and BAT.

### Bromodomain and Extra-Terminal Domain Inhibitors

Importantly, in addition to the use of HATis and HDACis as anti-obesity and anti-diabetic agents, scientists have also begun to explore other therapeutic alternatives in the recent years. Aside from HATs and HDACs, the bromodomain and extra-terminal (BET) domain family proteins (BRD2, BRD3, BRD4, and BRDT), which consists of two tandem bromodomains (BD1 and BD2) and an extra-terminal domain, are also involved in epigenetic-regulated gene transcription through interactions between the bromodomains and acetylated lysine residues on histones during cellular differentiation (136). As such, compounds which interfere with the activity of the bromodomain, and as a consequence, repress the transcriptional network, serve as attractive therapeutic agents to treat a variety of diseases, including metabolic-associated disorders.

A prominent example is the BET inhibitor (BETi), JQ1, which is the first selective inhibitor of BET domain family proteins (137). Notably, JQ1 has recently been demonstrated to impede adipogenesis (138). In response to JQ1 treatment, 3T3-L1 cells exhibited a decrease in intracellular lipid accumulation and expression of adipogenic genes (*Ppar*γ, *C/EBP*α, *Fabp4*, and *Adipsin*). Additionally, upon treatment with two other BETis, namely LY294002 (which binds to BD1) and RVX-208 (which binds to BD2), adipogenesis was inhibited as observed from the reduction in intracellular lipid accumulation. Since JQ1 binds to both BD1 and BD2, it can be deduced that blockade of either of the bromodomains interferes with adipogenesis. Subsequently, it was further revealed that 6 days after induction of differentiation, the expression of PPARγ and STAT5, as well as STAT5 phosphorylation were blunted. In addition, PPARγ and STAT5 target gene expression was also significantly affected (*Fabp4, Adipsin*, and *Myc, CIS, Pdk4, Fasn*, respectively). More importantly, *Ucp*1 expression was remarkably downregulated in JQ1-treated 3T3-L1 cells as compared to 3T3-L1 cells grown in brown fat conditions (T3), despite similar mitochondrial abundance. This indicates that JQ1 suppresses BAT differentiation and does not promote WAT browning. Similarly, in stromal vascular cells isolated from iBAT treated with JQ1, adipogenesis was inhibited. All in all, findings from this study implicate the use of JQ1, LY294002, and RVX-208 as potential anti-obesity drugs that inhibit both brown and white adipogenesis through the downregulation of adipogenic genes.

Recently, through *in vitro* and *in vivo* methods, BRD4 has also been identified to be essential for adipogenesis and myogenesis, where it functions as an enhancer epigenomic reader and interacts with active enhancers to stimulate the expression of cell-type-specific genes (139). Of note, Lee et al. showed that in *Brd4* conditional knockout mice, absence of *Brd4* resulted in a significant decrease in BAT and muscle mass (139). This implies that Brd4 is required for the development of BAT and muscle *in vivo*. On the other hand, in preadipocytes isolated from BAT, loss of *Brd4* lead to suppression of adipocyte identity gene expression (*Pparg, Cebpa*, and *Fabp4*) and adipogenesis. Additionally, in C2C12 myoblast, deletion of *Brd4* downregulated the expression of myogenesis identity genes and impaired myogenesis. These data show that Brd4 promotes the expression of genes associated with cell identity during the two processes. Gene ontology (GO) analyses further revealed that in particular, Brd4-dependent genes that were upregulated during adipogenesis, are related to adipocyte differentiation and lipid metabolism; while those that were upregulated during myogenesis are related to muscle development and function. In addition, it was found that *Brd2* and *Brd3* expression levels were upregulated in *Brd4* knockout preadipocytes. When treated with the BETi, JQ1, PPARγ-induced adipogenesis was completely defective, and on top of that, Rosi-stimulated PPARγ target gene expression was blocked. These findings imply that BRD4 functions redundantly with BRD2 and BRD3 in facilitating PPARγ-downstream adipogenic gene expression (139). In summary, the study demonstrated that BRD4 is critical for adipogenesis and myogenesis, and thus, could serve as a potential target for the treatment of obesity and its associated metabolic complications. Therefore, the development of BRD4-selective inhibitors (which impair adipocyte differentiation and adipose tissue development) as an alternative therapeutic approach to counteract obesity, holds therapeutic potential and remains an attractive field to be explored.

### ZnF-UBD Inhibitors

Ferreira de Freitas et al. recently revealed a novel pharmacological approach to inhibit HDAC6 catalytic activity through the use of small molecules that target the ZnF-UBD (Figure 6A) to prevent the recruitment of ubiquitinated protein aggregates (116, 117, 140). Further virtual screening and structural studies revealed inhibitor-induced conformational changes of the enzyme that led to the opening of a secondary cavity next to the primary binding pocket (PDB code: **5WBN**) (116). This cavity, along with the ZnF-UBD, can thus be exploited for the development of a new class of HDAC6-selective inhibitors with enhanced potency (Figure 6B).

### Non-histone Targets of HATs and HDACs

HATs and HDACs do not only target histone proteins. In the recent years, there is increasing research on the acetylation and deacetylation of non-histone proteins by HATs and HDACs, respectively (141). Many of these proteins are involved in key cellular processes such as stabilization of mRNA, protein localization and protein-protein interactions (142), and thus associated with diseases such as cancer (142), as well as neurological, inflammatory, immune, and metabolic disorders (143). As such, HATis and HDACis targeting these proteins are also implicated in various clinical therapies. For instance, it was reported that in response to nutrient starvation, SIRT1 deacetylates the non-histone protein, LC3 (143). Subsequently, this induces the interaction between LC3 and the diabetes and obesity-regulated gene (DOR). As a consequence, LC3 is transported from the nucleus to the cytoplasm where it associates with ATG7 to induce autophagy (143). In another study, it was unraveled that SIRT1 deacetylates the 66-kDa Src homology 2 domain-containing protein (p66Shc), resulting in the reduction of p66Shc-induced oxidative stress (144). Through the use of diabetic mice, it was further revealed that p66Shc acetylation on Lys81 leads to diabetic vascular endothelial dysfunction. These findings suggest that SIRT1 might have therapeutic potential in ameliorating diabetic vascular oxidative stress and vascular dysfunction (144).

### Future Directions

Finally, despite the potential therapeutic value of current HATis and HDACis, there still remain several questions to explore in future regarding the different mechanisms and processes surrounding thermogenic adipocyte differentiation and adaptive thermogenesis. For instance, in the cellular context, further studies should be carried out to investigate the crosstalk between histone acetylation and other histone modifications or other epigenetic mechanisms. Additionally, the interrelationship between histone acetylation and other cellular signaling cascades or molecules remains to be further delineated. In the pharmacological context, further advances to improve the selectivity and specificity of HATis and HDACis (i.e., for specific types of adipocytes, adipose tissues, HATs, and HDACs) could also enhance the potency and efficacy of the compounds. Moreover, since obesity can be classified into several categories, the therapeutic use of HATis and HDACis to specifically target the different stages of the thermogenic adipocyte differentiation process and BAT development would also be an interesting direction for scientists to look into. And last but not least, further efforts relating to drug repurposing in metabolic diseases would also be an alternative strategy as a means to improve current treatments and clinical outcomes for obesity and its associated metabolic comorbidities. To conclude, there seems to be a balance between HATs and HDACs to establish pro- and anti- adipogenic/thermogenic signals in response to specific environmental, nutritional, and cellular stimuli. In order for researchers to intervene and develop effective therapeutic strategies, one of the biggest challenges thus far would be to understand the exact contribution of the different players better.

## Author Contributions

BO, RB, and FX conceptualized and designed the review. BO wrote the manuscript and prepared the figures with essential input from RB, QZ, XP, MI, CL, and FX. All authors read and revised the manuscript.

### Conflict of Interest

The authors declare that the research was conducted in the absence of any commercial or financial relationships that could be construed as a potential conflict of interest.

## References

[1] Hales CM Carroll MD Fryar CD Ogden CL. Prevalence of obesity among adults and youth: United States, 2015-2016. NCHS Data Brief.(2017) 1–8. Available online at: https://www.cdc.gov/nchs/data/databriefs/db288.pdf 29155689

[2] Biener A Cawley J Meyerhoefer C. The impact of obesity on medical care costs and labor market outcomes in the US. Clin Chem. (2018) 64:108–17. 10.1373/clinchem.2017.272450 29097513

[3] World Health Organization Global patterns of health risk. Global Health Risks: Mortality and Burden of Disease Attributable to Selected Major Risks. Geneva: World Health Organization (2009). p. 9–12.

[4] Barnes AS. The epidemic of obesity and diabetes: trends and treatments. Tex Heart Inst J. (2011) 38:142–4. 21494521 PMC3066828

[5] Praso S Jusupovic F Ramic E Gledo I Ferkovic V Novakovic B. Obesity as a risk factor for artherial hypertension. Mater Sociomed. (2012) 24:87–90. 10.5455/msm.2012.24.87-90 23678314 PMC3633377

[6] Jiang SZ Lu W Zong XF Ruan HY Liu Y. Obesity and hypertension. Exp Ther Med. (2016) 12:2395–99. 10.3892/etm.2016.3667 27703502 PMC5038894

[7] Poirier P Giles TD Bray GA Hong Y Stern JS Pi-Sunyer FX. Obesity and cardiovascular disease: pathophysiology, evaluation, and effect of weight loss. Arterioscler Thromb Vasc Biol. (2006) 26:968–76. 10.1161/CIRCULATIONAHA.106.171016 16627822

[8] Calle EE Kaaks R. Overweight, obesity and cancer: epidemiological evidence and proposed mechanisms. Nat Rev Cancer. (2004) 4:579–91. 10.1038/nrc1408 15286738

[9] de Pergola G Silvestris F. Obesity as a major risk factor for cancer. J Obes. (2013) 2013:291546. 10.1155/2013/291546 24073332 PMC3773450

[10] Whittle AJ López M Vidal-Puig A. Using brown adipose tissue to treat obesity – the central issue. Trends Mol Med. (2011) 17:405–11. 10.1016/j.molmed.2011.04.001 21602104

[11] Betz MJ Enerbäck S. Targeting thermogenesis in brown fat and muscle to treat obesity and metabolic disease. Nat Rev Endocrinol. (2018) 14:77–87. 10.1038/nrendo.2017.132 29052591

[12] Townsend K Tseng Y-H. Brown adipose tissue: recent insights into development, metabolic function and therapeutic potential. Adipocyte. (2012) 1:13–24. 10.4161/adip.18951 23700507 PMC3661118

[13] Cypess AM Lehman S Williams G Tal I Rodman D Goldfine AB. Identification and importance of brown adipose tissue in adult humans. N Engl J Med. (2009) 360:1509–17. 10.1056/NEJMoa0810780 19357406 PMC2859951

[14] Choe SS Huh JY Hwang IJ Kim JI Kim JB. Adipose tissue remodeling: its role in energy metabolism and metabolic disorders. Front Endocrinol. (2016) 7:30. 10.3389/fendo.2016.00030 27148161 PMC4829583

[15] Stephens JM. The fat controller: adipocyte development. PLoS Biol. (2012) 10:e1001436. 10.1371/journal.pbio.1001436 23209380 PMC3507952

[16] Gupta RK. Adipocytes. Curr Biol. (2014) 24:R988–93. 10.1016/j.cub.2014.09.003 25442852

[17] Paulo E Wu D Wang Y Zhang Y Wu Y Swaney DL. Sympathetic inputs regulate adaptive thermogenesis in brown adipose tissue through cAMP-Salt inducible kinase axis. Sci Rep. (2018) 8:11001. 10.1038/s41598-018-29333-6 30030465 PMC6054673

[18] Porter C. Quantification of UCP1 function in human brown adipose tissue. Adipocyte. (2017) 6:167–74. 10.1080/21623945.2017.1319535 28453364 PMC5477712

[19] Wu J Bostrom P Sparks LM Ye L Choi JH Giang A-H. Beige adipocytes are a distinct type of thermogenic fat cell in mouse and human. Cell. (2012) 150:366–76. 10.1016/j.cell.2012.05.016 22796012 PMC3402601

[20] Wang Q Kajimura S. Naa10P puts a brake on PGC1alpha and fat browning. Nat Struct Mol Biol. (2019) 26:849–51. 10.1038/s41594-019-0310-2 31527714 PMC6815931

[21] Ikeda K Maretich P Kajimura S. The common and distinct features of brown and beige adipocytes. Trends Endocrinol Metab. (2018) 29:191–200. 10.1016/j.tem.2018.01.001 29366777 PMC5826798

[22] Vitali A Murano I Zingaretti MC Frontini A Ricquier D Cinti S. The adipose organ of obesity-prone C57BL/6J mice is composed of mixed white and brown adipocytes. J Lipid Res. (2012) 53:619–29. 10.1194/jlr.M018846 22271685 PMC3307639

[23] Dolinoy DC Jirtle RL. Environmental epigenomics in human health and disease. Environ Mol Mutagen. (2008) 49:4–8. 10.1002/em.20366 18172876

[24] Arif M Sadayappan S Becker RC Martin LJ Urbina EM. Epigenetic modification: a regulatory mechanism in essential hypertension. Hypertens Res. (2019) 42:1099–113. 10.1038/s41440-019-0248-0 30867575

[25] Martínez JA Milagro FI Claycombe KJ Schalinske KL. Epigenetics in adipose tissue, obesity, weight loss, and diabetes. Adv Nutr. (2014) 5:71–81. 10.3945/an.113.004705 24425725 PMC3884103

[26] Pham TX Lee J. Dietary regulation of histone acetylases and deacetylases for the prevention of metabolic diseases. Nutrients. (2012) 4:1868–86. 10.3390/nu4121868 23363995 PMC3546612

[27] Wang J Wu Z Li D Li N Dindot SV Satterfield MC. Nutrition, epigenetics, and metabolic syndrome. Antioxid Redox Signal. (2012) 17:282–301. 10.1089/ars.2011.4381 22044276 PMC3353821

[28] Gillberg L Hjort L Epigenetics of metabolic diseases. In: Tollefsbol TO, editor. Handbook of Epigenetics: The New Molecular and Medical Genetics. 2nd ed London: Academic Press (2017). p. 569–80. 10.1016/B978-0-12-805388-1.00037-7

[29] Li HX Xiao L Wang C Gao JL Zhai YG. Epigenetic regulation of adipocyte differentiation and adipogenesis. J Zhejiang Univ Sci B. (2010) 11:784–91. 10.1631/jzus.B0900401 20872986 PMC2950241

[30] Peng X Zhang Q Liao C Han W Xu F. Epigenomic control of thermogenic adipocyte differentiation and function. Int J Mol Sci. (2018) 19:E1793. 10.3390/ijms19061793 29914202 PMC6032041

[31] Portela A Esteller M. Epigenetic modifications and human disease. Nat Biotechnol. (2010) 28:1057–68. 10.1038/nbt.1685 20944598

[32] Scott RH Moore GE Epigenetic mechanisms of human imprinting disorders. In: Tollefsbol TO, editor. Epigenetics in Human Disease. 1st ed London: Academic Press (2012). p. 253–71. 10.1016/B978-0-12-388415-2.00013-5

[33] El-Osta A Wolffe AP. DNA methylation and histone deacetylation in the control of gene expression: basic biochemistry to human development and disease. Gene Expr. (2000) 9:63–75. 10.3727/000000001783992731 11097425 PMC5964960

[34] Suzuki H Maruyama R Yamamoto E Niinuma T Kai M. Relationship between noncoding RNA dysregulation and epigenetic mechanisms in cancer. In: Song E, editor. The Long and Short Non-coding RNAs in Cancer Biology Advances in Experimental Medicine and Biology, vol 927. Singapore: Springer (2016). p. 109–35. 10.1007/978-981-10-1498-7_4 27376733

[35] Schwartz YB Pirrotta V. Polycomb complexes and epigenetic states. Curr Opin Cell Biol. (2008) 20:266–73. 10.1016/j.ceb.2008.03.002 18439810

[36] Zhang Q Ramlee MK Brunmeir R Villanueva CJ Halperin D Xu F. Dynamic and distinct histone modifications modulate the expression of key adipogenesis regulatory genes. Cell Cycle. (2012) 11:4310–22. 10.4161/cc.22224 23085542 PMC3552913

[37] Brunmeir R Wu J Peng X Kim SY Julien SG Zhang Q. Comparative transcriptomic and epigenomic analyses reveal new regulators of murine brown adipogenesis. PLoS Genet. (2016) 12:e1006474. 10.1371/journal.pgen.1006474 27923061 PMC5140063

[38] Yoo EJ Chung JJ Choe SS Kim KH Kim JB. Down-regulation of histone deacetylases stimulates adipocyte differentiation. J Biol Chem. (2006) 281:6608–15. 10.1074/jbc.M508982200 16407282

[39] Lee JE Schmidt H Lai B Ge K. Transcriptional and epigenomic regulation of adipogenesis. Mol Cell Biol. (2019) 39:e00601-18. 10.1128/MCB.00601-18 30936246 PMC6517598

[40] Zhou Y Peng J Jiang S. Role of histone acetyltransferases and histone deacetylases in adipocyte differentiation and adipogenesis. Eur J Cell Biol. (2014) 93:170–7. 10.1016/j.ejcb.2014.03.001 24810880

[41] Gallinari P Di Marco S Jones P Pallaoro M Steinkühler C. HDACs, histone deacetylation and gene transcription: from molecular biology to cancer therapeutics. Cell Res. (2007) 17:195–211. 10.1038/sj.cr.7310149 17325692

[42] Allis CD Jenuwein T. The molecular hallmarks of epigenetic control. Nat Rev Genet. (2016) 17:487–500. 10.1038/nrg.2016.59 27346641

[43] Bannister AJ Kouzarides T. Regulation of chromatin by histone modifications. Cell Res. (2011) 21:381–95. 10.1038/cr.2011.22 21321607 PMC3193420

[44] Verdin E Ott M. 50 years of protein acetylation: from gene regulation to epigenetics, metabolism and beyond. Nat Rev Mol Cell Biol. (2015) 16:258–64. 10.1038/nrm3931 25549891

[45] Trisciuoglio D Di Martile M Del Bufalo D. Emerging role of histone acetyltransferase in stem cells and cancer. Stem Cells Int. (2018) 2018:8908751. 10.1155/2018/8908751 30651738 PMC6311713

[46] Ramlee MK Zhang Q Idris M Peng X Sim CK Han W. Histone H3 K27 acetylation marks a potent enhancer element on the adipogenic master regulator gene Pparg2. Cell Cycle. (2014) 13:3414–22. 10.4161/15384101.2014.953424 25485585 PMC4614625

[47] Xu F Zhang K Grunstein M. Acetylation in histone H3 globular domain regulates gene expression in yeast. Cell. (2005) 121:375–85. 10.1016/j.cell.2005.03.011 15882620

[48] Fang JY Lu YY. Effects of histone acetylation and DNA methylation on p21(WAF1) regulation. World J Gastroenterol. (2002) 8:400–5. 10.3748/wjg.v8.i3.400 12046058 PMC4656409

[49] Brownell JE Allis CD. Special HATs for special occasions: linking histone acetylation to chromatin assembly and gene activation. Curr Opin Genet Dev. (1996) 6:176–84. 10.1016/S0959-437X(96)80048-7 8722174

[50] Gräff J Tsai LH. Histone acetylation: molecular mnemonics on the chromatin. Nat Rev Neurosci. (2013) 14:97–111. 10.1038/nrn3427 23324667

[51] Li B Carey M Workman JL. The role of chromatin during transcription. Cell. (2007) 128:707–19. 10.1016/j.cell.2007.01.015 17320508

[52] Verdone L Agricola E Caserta M Di Mauro E. Histone acetylation in gene regulation. Brief Funct Genomic Proteomic. (2006) 5:209–21. 10.1093/bfgp/ell028 16877467

[53] Barnes PJ Adcock IM Ito K. Histone acetylation and deacetylation: importance in inflammatory lung diseases. Eur Respir J. (2005) 25:552–63. 10.1183/09031936.05.00117504 15738302

[54] Ren J Panther E Liao X Grammer AC Lipsky PE Reilly CM. The impact of protein acetylation/deacetylation on systemic lupus erythematosus. Int J Mol Sci. (2018) 19:4007. 10.3390/ijms19124007 30545086 PMC6321219

[55] Berndsen CE Denu JM. Catalysis and substrate selection by histone/protein lysine acetyltransferases. Curr Opin Struct Biol. (2008) 18:682–9. 10.1016/j.sbi.2008.11.004 19056256 PMC2723715

[56] Haberland M Montgomery RL Olson EN. The many roles of histone deacetylases in development and physiology: implications for disease and therapy. Nat Rev Genet. (2009) 10:32–42. 10.1038/nrg2485 19065135 PMC3215088

[57] Li W Sun Z. Mechanism of action for HDAC inhibitors—insights from omics approaches. Int J Mol Sci. (2019) 20:1616. 10.3390/ijms20071616 30939743 PMC6480157

[58] Audia JE Campbell RM. Histone modifications and cancer. Cold Spring Harb Perspect Biol. (2016) 8:a019521. 10.1101/cshperspect.a019521 27037415 PMC4817802

[59] Ropero S Esteller M. The role of histone deacetylases (HDACs) in human cancer. Mol Oncol. (2007) 1:19–25. 10.1016/j.molonc.2007.01.001 19383284 PMC5543853

[60] Di Cerbo V Schneider R. Cancers with wrong HATs: the impact of acetylation. Brief Funct Genomics. (2013) 12:231–43. 10.1093/bfgp/els065 23325510

[61] Lu X Wang L Yu C Yu D Yu G. Histone acetylation modifiers in the pathogenesis of Alzheimer's disease. Front Cell Neurosci. (2015) 9:226. 10.3389/fncel.2015.00226 26136662 PMC4468862

[62] Bonnaud EM Suberbielle E Malnou CE. Histone acetylation in neuronal (dys)function. Biomol Concepts. (2016) 7:103–16. 10.1515/bmc-2016-0002 27101554

[63] Park G Tan J Garcia G Kang Y Salvesen G Zhang Z. Regulation of histone acetylation by autophagy in Parkinson disease. J Biol Chem. (2016) 291:3531–40. 10.1074/jbc.M115.675488 26699403 PMC4751393

[64] Araki Y Mimura T. The histone modification code in the pathogenesis of autoimmune diseases. Mediators Inflamm. (2017) 2017:2608605. 10.1155/2017/2608605 28127155 PMC5239974

[65] Huber LC Brock M Hemmatazad H Giger OT Moritz F Trenkmann M. Histone deacetylase/acetylase activity in total synovial tissue derived from rheumatoid arthritis and osteoarthritis patients. Arthritis Rheum. (2007) 56:1087–93. 10.1002/art.22512 17393417

[66] Liu XY Xu JF. Reduced histone H3 acetylation in CD4(+) T lymphocytes: potential mechanism of latent autoimmune diabetes in adults. Dis Markers. (2015) 2015:285125. 10.1155/2015/285125 26839444 PMC4709642

[67] Wang Y Yang Y Luo Y Yin Y Wang Q Li Y. Aberrant histone modification in peripheral blood B cells from patients with systemic sclerosis. Clin Immunol. (2013) 149:46–54. 10.1016/j.clim.2013.06.006 23891737

[68] Kaczmarska Z Ortega E Goudarzi A Huang H Kim S Márquez JA. Structure of p300 in complex with acyl-CoA variants. Nat Chem Biol. (2017) 13:21–9. 10.1038/nchembio.2217 27820805 PMC5757799

[69] Schrodinger LLC The PyMOL Molecular Graphics System, Version 2.3.2 (2019).

[70] Lee CY Grant PA Chapter 1-1 - role of histone acetylation and acetyltransferases in gene regulation. In: McCullough SD Dolinoy DC, editors. Toxicoepigenetics. London: Academic Press (2019). p. 3–30. 10.1016/B978-0-12-812433-8.00001-0

[71] Marmorstein R. Structure of histone acetyltransferases. J Mol Biol. (2001) 311:433–44. 10.1006/jmbi.2001.4859 11492997

[72] Favrot L Blanchard JS Vergnolle O. Bacterial GCN5-related N-acetyltransferases: from resistance to regulation. Biochemistry. (2016) 55:989–1002. 10.1021/acs.biochem.5b01269 26818562 PMC4795176

[73] Salah Ud-Din AIM Tikhomirova A Roujeinikova A Structure and functional diversity of GCN5-related N-acetyltransferases (GNAT). Int J Mol Sci. (2016) 17:1018 10.3390/ijms17071018 PMC4964394 27367672

[74] Mikula M Majewska A Ledwon JK Dzwonek A Ostrowski J. Obesity increases histone H3 lysine 9 and 18 acetylation at Tnfa and Ccl2 genes in mouse liver. Int J Mol Med. (2014) 34:1647–54. 10.3892/ijmm.2014.1958 25319795

[75] Miao F Gonzalo IG Lanting L Natarajan R. *In vivo* chromatin remodeling events leading to inflammatory gene transcription under diabetic conditions. J Biol Chem. (2004) 279:18091–7. 10.1074/jbc.M311786200 14976218

[76] Panchenko PE Voisin S Jouin M Jouneau L Prézelin A Lecoutre S. Expression of epigenetic machinery genes is sensitive to maternal obesity and weight loss in relation to fetal growth in mice. Clin Epigenetics. (2016) 8:22. 10.1186/s13148-016-0188-3 26925174 PMC4769534

[77] Dorneles GP Boeira MCR Schipper LL Silva IRV Elsner VR Dal Lago P. Acute strenuous exercise induces an imbalance on histone H4 acetylation/histone deacetylase 2 and increases the proinflammatory profile of PBMC of obese individuals. Oxid Med Cell Longev. (2017) 2017:1530230. 10.1155/2017/1530230 29142617 PMC5671743

[78] Jin Q Yu LR Wang L Zhang Z Kasper LH Lee JE. Distinct roles of GCN5/PCAF-mediated H3K9ac and CBP/p300-mediated H3K18/27ac in nuclear receptor transactivation. EMBO J. (2011) 30:249–62. 10.1038/emboj.2010.318 21131905 PMC3025463

[79] Jin Q Wang C Kuang X Feng X Sartorelli V Ying H. Gcn5 and PCAF regulate PPARγ and Prdm16 expression to facilitate brown adipogenesis. Mol Cell Biol. (2014) 34:3746–53. 10.1128/MCB.00622-14 25071153 PMC4187735

[80] Steger DJ Grant GR Schupp M Tomaru T Lefterova MI Schug J. Propagation of adipogenic signals through an epigenomic transition state. Genes Dev. (2010) 24:1035–44. 10.1101/gad.1907110 20478996 PMC2867208

[81] Schuetz A Bernstein G Dong A Antoshenko T Wu H Loppnau P. Crystal structure of a binary complex between human GCN5 histone acetyltransferase domain and acetyl coenzyme A. Proteins. (2007) 68:403–7. 10.1002/prot.21407 17410582

[82] Clements A Rojas JR Trievel RC Wang L Berger SL Marmorstein R. Crystal structure of the histone acetyltransferase domain of the human PCAF transcriptional regulator bound to coenzyme A. EMBO J. (1999) 18:3521–32. 10.1093/emboj/18.13.3521 10393169 PMC1171431

[83] Shi S Lin J Cai Y Yu J Hong H Ji K. Dimeric structure of p300/CBP associated factor. BMC Struct Biol. (2014) 14:2. 10.1186/1472-6807-14-2 24423233 PMC3897949

[84] Lai B Lee JE Jang Y Wang L Peng W Ge K. MLL3/MLL4 are required for CBP/p300 binding on enhancers and super-enhancer formation in brown adipogenesis. Nucleic Acids Res. (2017) 45:6388–403. 10.1093/nar/gkx234 28398509 PMC5499743

[85] Takahashi N Kawada T Yamamoto T Goto T Taimatsu A Aoki N. Overexpression and ribozyme-mediated targeting of transcriptional coactivators CREB-binding protein and p300 revealed their indispensable roles in adipocyte differentiation through the regulation of peroxisome proliferator-activated receptor gamma. J Biol Chem. (2002) 277:16906–12. 10.1074/jbc.M200585200 11884404

[86] Liu X Wang L Zhao K Thompson PR Hwang Y Marmorstein R. The structural basis of protein acetylation by the p300/CBP transcriptional coactivator. Nature. (2008) 451:846–50. 10.1038/nature06546 18273021

[87] Delvecchio M Gaucher J Aguilar-Gurrieri C Ortega E Panne D. Structure of the p300 catalytic core and implications for chromatin targeting and HAT regulation. Nat Struct Mol Biol. (2013) 20:1040–6. 10.1038/nsmb.2642 23934153

[88] Ortega E Rengachari S Ibrahim Z Hoghoughi N Gaucher J Holehouse AS. Transcription factor dimerization activates the p300 acetyltransferase. Nature. (2018) 562:538–44. 10.1038/s41586-018-0621-1 30323286 PMC6914384

[89] Maksimoska J Segura-Peña D Cole PA Marmorstein R. Structure of the p300 histone acetyltransferase bound to acetyl-coenzyme A and its analogues. Biochemistry. (2014) 53:3415–22. 10.1021/bi500380f 24819397 PMC4045318

[90] Sheppard HM Harries JC Hussain S Bevan C Heery DM. Analysis of the steroid receptor coactivator 1 (SRC1)-CREB binding protein interaction interface and its importance for the function of SRC1. Mol Cell Biol. (2001) 21:39–50. 10.1128/MCB.21.1.39-50.2001 11113179 PMC86566

[91] Picard F Géhin M Annicotte JS Rocchi S Champy MF O'Malley BW. SRC-1 and TIF2 control energy balance between white and brown adipose tissues. Cell. (2002) 111:931–41. 10.1016/S0092-8674(02)01169-8 12507421

[92] Wang Z Qi C Krones A Woodring P Zhu X Reddy JK. Critical roles of the p160 transcriptional coactivators p/CIP and SRC-1 in energy balance. Cell Metab. (2006) 3:111–22. 10.1016/j.cmet.2006.01.002 16459312

[93] Balasubramanyam K Varier RA Altaf M Swaminathan V Siddappa NB Ranga U. Curcumin, a novel p300/CREB-binding protein-specific inhibitor of acetyltransferase, represses the acetylation of histone/nonhistone proteins and histone acetyltransferase-dependent chromatin transcription. J Biol Chem. (2004) 279:51163–71. 10.1074/jbc.M409024200 15383533

[94] Weisberg SP Leibel R Tortoriello DV. Dietary curcumin significantly improves obesity-associated inflammation and diabetes in mouse models of diabesity. Endocrinology. (2008) 149:3549–58. 10.1210/en.2008-0262 18403477 PMC2453081

[95] Shao W Yu Z Chiang Y Yang Y Chai T Foltz W. Curcumin prevents high fat diet induced insulin resistance and obesity via attenuating lipogenesis in liver and inflammatory pathway in adipocytes. PLoS ONE. (2012) 7:e28784. 10.1371/journal.pone.0028784 22253696 PMC3253779

[96] Wang Y Wang Y Luo M Wu H Kong L Xin Y. Novel curcumin analog C66 prevents diabetic nephropathy via JNK pathway with the involvement of p300/CBP-mediated histone acetylation. Biochim Biophys Acta. (2015) 1852:34–46. 10.1016/j.bbadis.2014.11.006 25446993 PMC4369325

[97] de Marinis Y Cai M Bompada P Atac D Kotova O Johansson ME. Epigenetic regulation of the thioredoxin-interacting protein (TXNIP) gene by hyperglycemia in kidney. Kidney Int. (2016) 89:342–53. 10.1016/j.kint.2015.12.018 26806835

[98] Choi KC Jung MG Lee Y-H Yoon JC Kwon SH Kang HB. Epigallocatechin-3-gallate, a histone acetyltransferase inhibitor, inhibits EBV-induced B lymphocyte transformation via suppression of RelA acetylation. Cancer Res. (2009) 69:583–92. 10.1158/0008-5472.CAN-08-2442 19147572

[99] Klaus S Pültz S Thöne-Reineke C Wolfram S. Epigallocatechin gallate attenuates diet-induced obesity in mice by decreasing energy absorption and increasing fat oxidation. Int J Obes. (2005) 29:615–23. 10.1038/sj.ijo.0802926 15738931

[100] Zhou J Mao L Xu P Wang Y. Effects of (-)-epigallocatechin gallate (EGCG) on energy expenditure and microglia-mediated hypothalamic inflammation in mice fed a high-fat diet. Nutrients. (2018) 10:1681. 10.3390/nu10111681 30400620 PMC6266769

[101] Li F Gao C Yan P Zhang M Wang Y Hu Y. EGCG reduces obesity and white adipose tissue gain partly through AMPK activation in mice. Front Pharmacol. (2018) 9:1366. 10.3389/fphar.2018.01366 30524290 PMC6262053

[102] Barszcz M Taciak M Tuśnio A Skomial J. Effects of dietary level of tannic acid and protein on internal organ weights and biochemical blood parameters of rats. PLoS ONE. (2018) 13:e0190769. 10.1371/journal.pone.0190769 29304153 PMC5755905

[103] Liu X Kim JK Li Y Li J Liu F Chen X. Tannic acid stimulates glucose transport and inhibits adipocyte differentiation in 3T3-L1 cells. J Nutr. (2005) 135:165–71. 10.1093/jn/135.2.165 15671208

[104] Ong KC Khoo HE Das NP. Tannic acid inhibits insulin-stimulated lipogenesis in rat adipose tissue and insulin receptor function *in vitro*. Experientia. (1995) 51:577–84. 10.1007/BF02128747 7607300

[105] Nie F Liang Y Xun H Sun J He F Ma X. Inhibitory effects of tannic acid in the early stage of 3T3-L1 preadipocytes differentiation by down-regulating PPARgamma expression. Food Funct. (2015) 6:894–901. 10.1039/C4FO00871E 25623997

[106] Chung MY Song JH Lee J Shin EJ Park JH Lee SH. Tannic acid, a novel histone acetyltransferase inhibitor, prevents non-alcoholic fatty liver disease both *in vivo* and *in vitro* model. Mol Metab. (2019) 19:34–48. 10.1016/j.molmet.2018.11.001 30473486 PMC6323241

[107] Marks PA Xu WS. Histone deacetylase inhibitors: potential in cancer therapy. J Cell Biochem. (2009) 107:600–8. 10.1002/jcb.22185 19459166 PMC2766855

[108] Korfei M Skwarna S Henneke I MacKenzie B Klymenko O Saito S. Aberrant expression and activity of histone deacetylases in sporadic idiopathic pulmonary fibrosis. Thorax. (2015) 70:1022–32. 10.1136/thoraxjnl-2014-206411 26359372

[109] Gao L Cueto MA Asselbergs F Atadja P. Cloning and functional characterization of HDAC11, a novel member of the human histone deacetylase family. J Biol Chem. (2002) 277:25748–55. 10.1074/jbc.M111871200 11948178

[110] Bürger M Chory J Structural and chemical biology of deacetylases for carbohydrates, proteins, small molecules and histones. Commun Biol. (2018) 1:217 10.1038/s42003-018-0214-4 30534609 PMC6281622

[111] Lombardi PM Cole KE Dowling DP Christianson DW. Structure, mechanism, and inhibition of histone deacetylases and related metalloenzymes. Curr Opin Struct Biol. (2011) 21:735–43. 10.1016/j.sbi.2011.08.004 21872466 PMC3232309

[112] Lauffer BE Mintzer R Fong R Mukund S Tam C Zilberleyb I Histone deacetylase (HDAC) inhibitor kinetic rate constants correlate with cellular histone acetylation but not transcription and cell viability. J Biol Chem. (2013) 288:26926–43. 10.1074/jbc.M113.490706 23897821 PMC3772242

[113] Finnin MS Donigian JR Pavletich NP. Structure of the histone deacetylase SIRT2. Nat Struct Biol. (2001) 8:621–5. 10.1038/89668 11427894

[114] Bottomley MJ Lo Surdo P Di Giovine P Cirillo A Scarpelli R Ferrigno F. Structural and functional analysis of the human HDAC4 catalytic domain reveals a regulatory structural zinc-binding domain. J Biol Chem. (2008) 283:26694–704. 10.1074/jbc.M803514200 18614528 PMC3258910

[115] Schuetz A Min J Allali-Hassani A Schapira M Shuen M Loppnau P. Human HDAC7 harbors a class IIa histone deacetylase-specific zinc binding motif and cryptic deacetylase activity. J Biol Chem. (2008) 283:11355–63. 10.1074/jbc.M707362200 18285338 PMC2431080

[116] Ferreira de Freitas R Harding RJ Franzoni I Ravichandran M Mann MK Ouyang H. Identification and structure-activity relationship of HDAC6 zinc-finger ubiquitin binding domain inhibitors. J Med Chem. (2018) 61:4517–27. 10.1021/acs.jmedchem.8b00258 29741882

[117] Ouyang H Ali YO Ravichandran M Dong A Qiu W MacKenzie F. Protein aggregates are recruited to aggresome by histone deacetylase 6 via unanchored ubiquitin C termini. J Biol Chem. (2012) 287:2317–27. 10.1074/jbc.M111.273730 22069321 PMC3268394

[118] Anderson KA Madsen AS Olsen CA Hirschey MD Metabolic control by sirtuins and other enzymes that sense NAD(+), NADH, or their ratio. Biochim Biophys Acta Bioenerg. (2017) 1858:991–8. 10.1016/j.bbabio.2017.09.005 28947253 PMC5648639

[119] Li F Wu R Cui X Zha L Yu L Shi H. Histone deacetylase 1 (HDAC1) negatively regulates thermogenic program in brown adipocytes via coordinated regulation of histone H3 lysine 27 (H3K27) deacetylation and methylation. J Biol Chem. (2016) 291:4523–36. 10.1074/jbc.M115.677930 26733201 PMC4813478

[120] Shinoda K Ohyama K Hasegawa Y Chang HY Ogura M Sato A. Phosphoproteomics identifies CK2 as a negative regulator of beige adipocyte thermogenesis and energy expenditure. Cell Metab. (2015) 22:997–1008. 10.1016/j.cmet.2015.09.029 26525534 PMC4670581

[121] Emmett MJ Lim HW Jager J Richter HJ Adlanmerini M Peed LC. Histone deacetylase 3 prepares brown adipose tissue for acute thermogenic challenge. Nature. (2017) 546:544–8. 10.1038/nature22819 28614293 PMC5826652

[122] Ferrari A Longo R Fiorino E Silva R Mitro N Cermenati G. HDAC3 is a molecular brake of the metabolic switch supporting white adipose tissue browning. Nat Commun. (2017) 8:93. 10.1038/s41467-017-00182-7 28733645 PMC5522415

[123] Liao J Jiang J Jun H Qiao X Emont MP Kim DI. HDAC3-selective inhibition activates brown and beige fat through PRDM16. Endocrinology. (2018) 159:2520–7. 10.1210/en.2018-00257 29757434 PMC6456926

[124] Jung S Han M Korm S Lee SI Noh S Phorl S. HDAC6 regulates thermogenesis of brown adipocytes through activating PKA to induce UCP1 expression. Biochem Biophys Res Commun. (2018) 503:285–90. 10.1016/j.bbrc.2018.06.016 29890133

[125] Sun L Marin de Evsikova C Bian K Achille A Telles E Pei H. Programming and regulation of metabolic homeostasis by HDAC11. EBioMedicine. (2018) 33:157–68. 10.1016/j.ebiom.2018.06.025 29958910 PMC6085537

[126] Bagchi RA Ferguson BS Stratton MS Hu T Cavasin MA Sun L. HDAC11 suppresses the thermogenic program of adipose tissue via BRD2. JCI Insight. (2018) 3:e120159. 10.1172/jci.insight.120159 30089714 PMC6129125

[127] Qiang L Wang L Kon N Zhao W Lee S Zhang Y. Brown remodeling of white adipose tissue by SirT1-dependent deacetylation of Ppargamma. Cell. (2012) 150:620–32. 10.1016/j.cell.2012.06.027 22863012 PMC3413172

[128] Boutant M Joffraud M Kulkarni SS Garcia-Casarrubios E Garcia-Roves PM Ratajczak J. SIRT1 enhances glucose tolerance by potentiating brown adipose tissue function. Mol Metab. (2015) 4:118–31. 10.1016/j.molmet.2014.12.008 25685699 PMC4314542

[129] Shi T Wang F Stieren E Tong Q. SIRT3, a mitochondrial sirtuin deacetylase, regulates mitochondrial function and thermogenesis in brown adipocytes. J Biol Chem. (2005) 280:13560–7. 10.1074/jbc.M414670200 15653680

[130] Galmozzi A Mitro N Ferrari A Gers E Gilardi F Godio C. Inhibition of class I histone deacetylases unveils a mitochondrial signature and enhances oxidative metabolism in skeletal muscle and adipose tissue. Diabetes. (2013) 62:732–42. 10.2337/db12-0548 23069623 PMC3581211

[131] Ferrari A Fiorino E Longo R Barilla S Mitro N Cermenati G. Attenuation of diet-induced obesity and induction of white fat browning with a chemical inhibitor of histone deacetylases. Int J Obes. (2017) 41:289–98. 10.1038/ijo.2016.191 27795551

[132] Ferrari A Longo R Peri C Coppi L Caruso D Mai A. Inhibition of class I HDACs imprints adipogenesis toward oxidative and brown-like phenotype. Biochim Biophys Acta Mol Cell Biol Lipids. (2020) 1865:158594. 10.1016/j.bbalip.2019.158594 31904421

[133] Khan S Kumar S Jena G. Valproic acid reduces insulin-resistance, fat deposition and FOXO1-mediated gluconeogenesis in type-2 diabetic rat. Biochimie. (2016) 125:42–52. 10.1016/j.biochi.2016.02.014 26944797

[134] Khan S Jena G. Sodium butyrate reduces insulin-resistance, fat accumulation and dyslipidemia in type-2 diabetic rat: a comparative study with metformin. Chem Biol Interact. (2016) 254:124–34. 10.1016/j.cbi.2016.06.007 27270450

[135] Gao Z Yin J Zhang J Ward RE Martin RJ Lefevre M. Butyrate improves insulin sensitivity and increases energy expenditure in mice. Diabetes. (2009) 58:1509–17. 10.2337/db08-1637 19366864 PMC2699871

[136] Taniguchi Y. The bromodomain and extra-terminal Domain (BET) family: functional anatomy of BET paralogous proteins. Int J Mol Sci. (2016) 17:1849. 10.3390/ijms17111849 27827996 PMC5133849

[137] Filippakopoulos P Qi J Picaud S Shen Y Smith WB Fedorov O. Selective inhibition of BET bromodomains. Nature. (2010) 468:1067–73. 10.1038/nature09504 20871596 PMC3010259

[138] Goupille O Penglong T Kadri Z Granger-Locatelli M Fucharoen S Maouche-Chrétien L. Inhibition of the acetyl lysine-binding pocket of bromodomain and extraterminal domain proteins interferes with adipogenesis. Biochem Biophys Res Commun. (2016) 472:624–30. 10.1016/j.bbrc.2016.03.013 26972250

[139] Lee JE Park YK Park S Jang Y Waring N Dey A. Brd4 binds to active enhancers to control cell identity gene induction in adipogenesis and myogenesis. Nat Commun. (2017) 8:2217. 10.1038/s41467-017-02403-5 29263365 PMC5738375

[140] Harding RJ Ferreira de Freitas R Collins P Franzoni I Ravichandran M Ouyang H. Small molecule antagonists of the interaction between the histone deacetylase 6 zinc-finger domain and ubiquitin. J Med Chem. (2017) 60:9090–96. 10.1021/acs.jmedchem.7b00933 29019676

[141] Kim E Bisson WH Löhr CV Williams DE Ho E Dashwood RH. Histone and non-histone targets of dietary deacetylase inhibitors. Curr Top Med Chem. (2016) 16:714–31. 10.2174/1568026615666150825125857 26303421 PMC5087604

[142] Singh BN Zhang G Hwa YL Li J Dowdy SC Jiang SW. Nonhistone protein acetylation as cancer therapy targets. Expert Rev Anticancer Ther. (2010) 10:935–54. 10.1586/era.10.62 20553216 PMC3273412

[143] Narita T Weinert BT Choudhary C Functions and mechanisms of non-histone protein acetylation. Nat Rev Mol Cell Biol. (2019) 20:156–74. 10.1038/s41580-018-0081-3 30467427

[144] Kumar S Kim YR Vikram A Naqvi A Li Q Kassan M. Sirtuin1-regulated lysine acetylation of p66Shc governs diabetes-induced vascular oxidative stress and endothelial dysfunction. Proc Natl Acad Sci USA. (2017) 114:1714–19. 10.1073/pnas.1614112114 28137876 PMC5321021

